# Complex trait architecture: the pleiotropic model revisited

**DOI:** 10.1038/srep09351

**Published:** 2015-03-20

**Authors:** T.-L. North, M. A. Beaumont

**Affiliations:** 1 MRC-funded PhD student in the Bristol Centre for Systems Biomedicine (BCSBmed) doctoral training centre, Bristol Genetic Epidemiology Laboratories, School of Social and Community Medicine, University of Bristol, Bristol, UK; 2 Department of Mathematics and School of Biological Sciences, University of Bristol, Bristol, UK

## Abstract

There is currently much debate about how much the genetic heritability of complex traits is due to very rare alleles. This issue is important because it determines sampling strategies for genetic association studies. Several recent theoretical papers based on a pleiotropic model for trait evolution suggest that it is possible that a large proportion of the genetic variance could be explained by rare alleles. This model assumes that mutations with a large effect on fitness also tend to have large positive or negative effects on phenotypic traits. We show that conclusions based on standard diffusion results are generally applicable to simulations of whole genomes with overlapping generations in a finite population, although the variance contribution of rare alleles is somewhat smaller than theoretical predictions. We show that under many scenarios the pleiotropic model predicts trait distributions that are unrealistically leptokurtic. We argue that this imposes a limit on the relationship between fitness and trait effects.

A widely sought goal in biology is to understand the genetic architecture underlying phenotypic variation. In human genetics a distinction is made between Mendelian traits, usually affected by rare genes of large effect, and complex traits where the genetic component of their variation depends on a number of polymorphisms at different locations in the genome. Genome-wide association studies (GWAS) have endeavoured to identify these locations, typically for traits associated with disease, yet by and large this has not been very successful in unequivocally identifying a set of specific mutations that explain most of the heritability for any particular trait. It would appear that there are many mutations involved, and the distribution of genetic effects of individual mutations attenuates rapidly so that even with large sample sizes only a small fraction can be identified with reasonable statistical certainty, and together these often explain only a small fraction of heritability: the famous “missing heritability” problem^1^. However, if the requirement to positively identify a set of specific mutations is dropped, then a large proportion of heritability can be predicted from individual genotypes with a sufficiently dense marker set^2^. Although these observations together indicate that a large number of mutations must be involved in causing genetic variation in most traits, there is still uncertainty whether these mutations are very rare but with a large effect, or more common and with small effect^3^. This uncertainty raises the possibility that variants with very low MAF (Minor Allele Frequency) but with large effect size could be contributing a large proportion of the genetic variance of complex traits.

A useful modelling approach for examining these issues is one in which deleterious mutations have random effects on a trait, the magnitude of which depends on their selective effect^4^,^5^,^6^. This joint distribution has been taken as bivariate Wishart^4^, where the effects on fitness can be viewed as representing collective effects of the mutation on traits other than that under study^7^. In a recent modification of this model by Eyre-Walker^6^ the deleterious selection coefficient is assumed to be drawn from a gamma distribution. The trait effect of the mutation, conditional on its selection coefficient, is drawn from a distribution that allows for either zero or increasing levels of correlation between the absolute value of the trait effect and the selective effect. With this parameterisation it is possible to derive an expression for the proportion of the genetic variance in the trait due to all mutations at a particular frequency^6^. This formulation allows the issue of missing heritability to be addressed inferentially in terms of the proportion of genetic variance that is contributed by common or rare alleles. The main finding is that unless the mean strength of selection is much weaker than the evidence suggests, or variance in the trait is independent of fitness, the genetic variance of a complex trait will be due to derived mutations at a very low allele frequency^6^. This lends support to the rare variant hypothesis for complex disease architecture and has implications for the design of future genotyping arrays and the necessary sample sizes. Recent studies^8^,^9^,^10^,^11^ have used forward-time simulations to examine different scenarios, and have generally concluded that rare alleles of large effect may, indeed, explain a large proportion of genetic variance, although this conclusion depends on the joint distribution of mutational effects on traits and fitness.

The present study is motivated by two concerns. An advantage of the method of Eyre-Walker is that it gives a useful expression for the density of the variance of a complex trait. However, a possible limitation of using a diffusion model is that an infinite population size is assumed and, potentially more importantly, all loci are assumed to be independent. We use a forward-time Moran model with infinite sites and free recombination to compare directly with the analytical results. A degree of linkage disequilibrium is generated in the model because selection acts on whole genomes rather than individual loci. We show that the variance contribution of low frequency alleles can be lower than previously suggested^6^. This, we suggest, may be due to Hill-Robertson interference^12^. This is an effect whereby there is a reduction in the effectiveness of selection on multiple loci due to association between positively and negatively selected alleles at different loci. The effect declines with an increasing recombination rate but is still present in unlinked genes, in which the recombination rate is 0.5^13^. In addition, we highlight that the trait distribution implied by this model is unrealistically leptokurtic, which we argue imposes an upper limit on the magnitude of the correlation between trait effects and selective effects.

## Results

### The model

We implemented a forward-time Moran model which incorporates the relationship between fitness and trait effects as previously described^6^. In this model there is no direct causal relationship between the effect of a mutation on fitness and the trait value. One justification of this is to imagine that mutations affect a large number of traits, some of which are components of fitness, and thus the fitness effect of any particular mutant represents the combined effect of a mutation on all traits other than that under study^7^. Details of our implementation are given in the Methods, and are similar to those in an earlier study^14^. The joint distribution is modelled^6^ by a gamma distribution for the scaled (deleterious) selection coefficient, *S* = 4*Ns*. The trait effect, *z*, is distributed as:
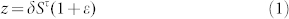
where *N* is the diploid population size, *ε* is a normally distributed noise term with mean 0, and *δ* takes the value 1 or −1 with equal probability. Depending on the scaling parameter, *τ*, *z* and *S* can be independent (*τ* = 0), or more strongly correlated with increasing *τ*. (Note, however, that *z* and *S* are uncorrelated – only the absolute magnitude of *z* depends on *S*.) This will be referred to below as the power function model. Further details of the computer simulation are provided in the Methods.

As described later, for many parameter values, the model of mutational effects given by (1) results in an equilibrium trait distribution across individuals that is highly leptokurtic. We therefore examined the consequences of an alternative conditional distribution for the trait effect given *S*, defined by:
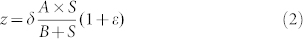
where all parameters are as defined in equation (1), but *τ* is replaced by parameters *A* and *B*, which characterize a saturating function that prevents extreme mutational effects on the trait. The motivation is that there are mechanical, or homeostatic, or developmental constraints that prevent extreme changes. For additional biological realism it might be preferable to apply such a saturating function to the overall trait value of an individual. However this would then imply a non-additive genetic model, which would make the interpretation of the results more complex. The term 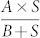, as in Michaelis Menten kinetics^15^, has gradient 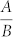 near the origin and approaches the limit *A* as *S* → 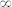. We refer to this model below as the saturating function model.

We implemented 5 different parameter combinations using the original conditional distribution and 3 different combinations using the proposed new function (Table 1). We present the relationship between *S* and *z* for each model in Supplementary Figure S19. In all models, we set haploid population size (2*N*) = 10,000, where *N* is the equivalent diploid population size, generation time = 1, simulation time = 60,000 generations, genome mutation rate *U* = 0.1, *β* (shape parameter of gamma distributed strength of selection) = 0.2^16^, mean *ε* = 0, standard deviation *ε* = 1. We chose 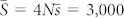 or 30 (Table 1), where 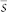 is the mean (deleterious) effect of mutations. Most parameter combinations are chosen to compare with those in the earlier study^6^. However, since we are additionally interested in the distribution of trait and fitness effects within the population, the choice of genomic mutation rate is important. We chose a value of 0.1 to give 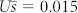 in the strong selection case and 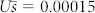 for weaker selection. The strong selection case can be compared with the range of estimates of 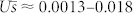 in heterozygotes assuming codominance^17^. The results presented here are based on 100 independent runs for each set of parameter combinations. It should be noted that in the pleiotropic model the evolution of the system depends only on the distribution of selective effects and not on the trait effects. Hence only parameter combinations that modify selection will be expected to change the distribution of fitness across individuals, and it is possible in principle to apply different phenotypic models to the same set of simulated data. However, to avoid spurious correlations, we chose to implement separate sets of simulations for all parameter combinations.

### The distribution of fitness and the trait

**Figure 1:**
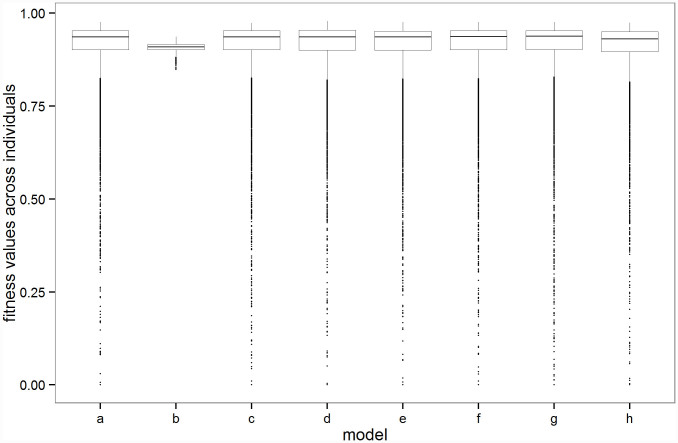
Distribution of fitness from run 1 of each model. These are representative of the 100 replicate runs with each parameter combination. The model parameters are given in Table 1. As explained in the text, with the exception of model b, the same evolutionary model is implemented and hence the distribution of fitness is expected to be the same. The whiskers extend from the 25^th^ and 75^th^ percentiles to values within 1.5 x the inter-quartile range^25^.

In simulations where the mean selective effect of mutations is high (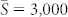) there is a highly skewed distribution of fitness within the population (Figure 1), where around 1.5% of individuals have fitness less than 0.5. By contrast, when the selective effect is lower (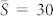; Figure 1 model b) the distribution of individual fitness is close to the maximum value of 1. The different distributions for strong and weaker selection are explained by noting that with a genomic mutation rate of 0.1, 1,000 new mutations are expected to arise in the population each generation. Prior to selection, new mutations for high or low 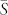 have a gamma distribution with 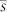 of respectively 0.15, and 0.0015, giving rise to the observed tails in the figures.

**Figure 2:**
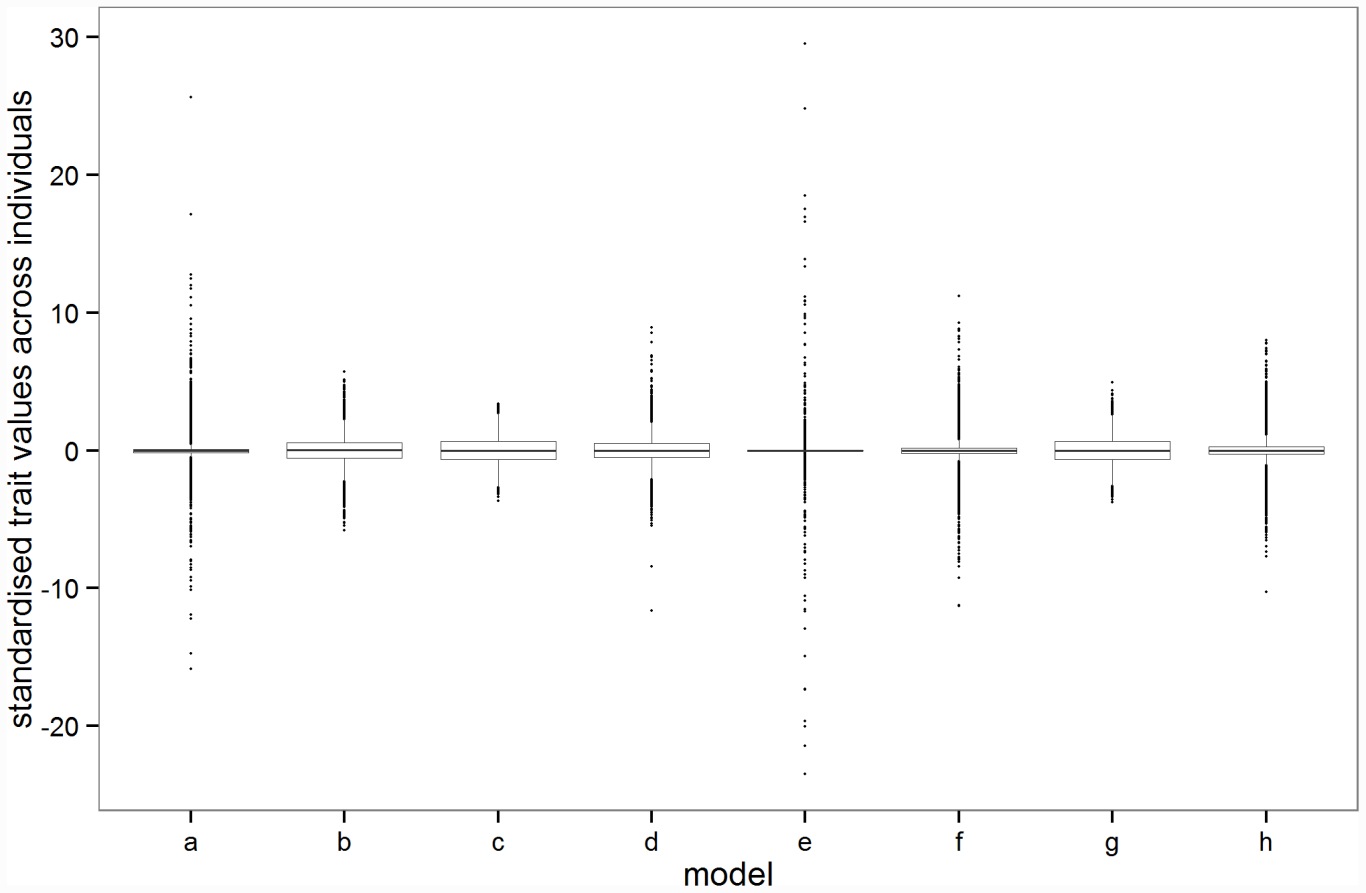
Distribution of the trait from run 1 of each model. These are representative of the 100 replicate runs with each parameter combination. The model parameters are given in Table 1. Trait values were standardised for each model by subtracting the mean and dividing by the standard deviation. The whiskers extend from the 25^th^ and 75^th^ percentiles to values within 1.5 x the inter-quartile range^25^.

The distribution of the trait across individuals in the population tends to be highly leptokurtic (Figure 2). The trait distributions represent the breeding values (genetic effects only) without environmental effects. Note that the raw scale in which the trait effects are measured is arbitrary, and depends on the formulation used in equations 1 and 2, following Eyre-Walker^6^, and is thus a (non-linear) function of the mean strength of selection. Kurtosis is most pronounced when the absolute trait effect is linearly or greater than linearly related to fitness (*τ* > = 1). When selection is weak (model b) or when the absolute trait effect and fitness are uncorrelated (model c), the trait distribution has less extreme tails. In our alternative model we find that when the mutational effects on fitness and absolute trait value are the same for small effect sizes (i.e. *A* and *B* are equal) the distributions are leptokurtic irrespective of a threshold of *A* = *B* = 10,000 or *A* = *B* = 4,000 (Figure 2, models f, h). However, when the mutational effect on the trait is much stronger than on fitness (Figure 2, model g; *A* = 4,000 and *B* = 40; gradient for small effects ~ 100) we see a more closely normal distribution, similar to the uncorrelated (*τ* = 0) case for the original model. In this latter model the trait effect associated with rare low-fitness variants is truncated and hence the effects of individual mutations on the trait are less leptokurtic than in the former two models.

### The genetic architecture of the trait

**Figure 3:**
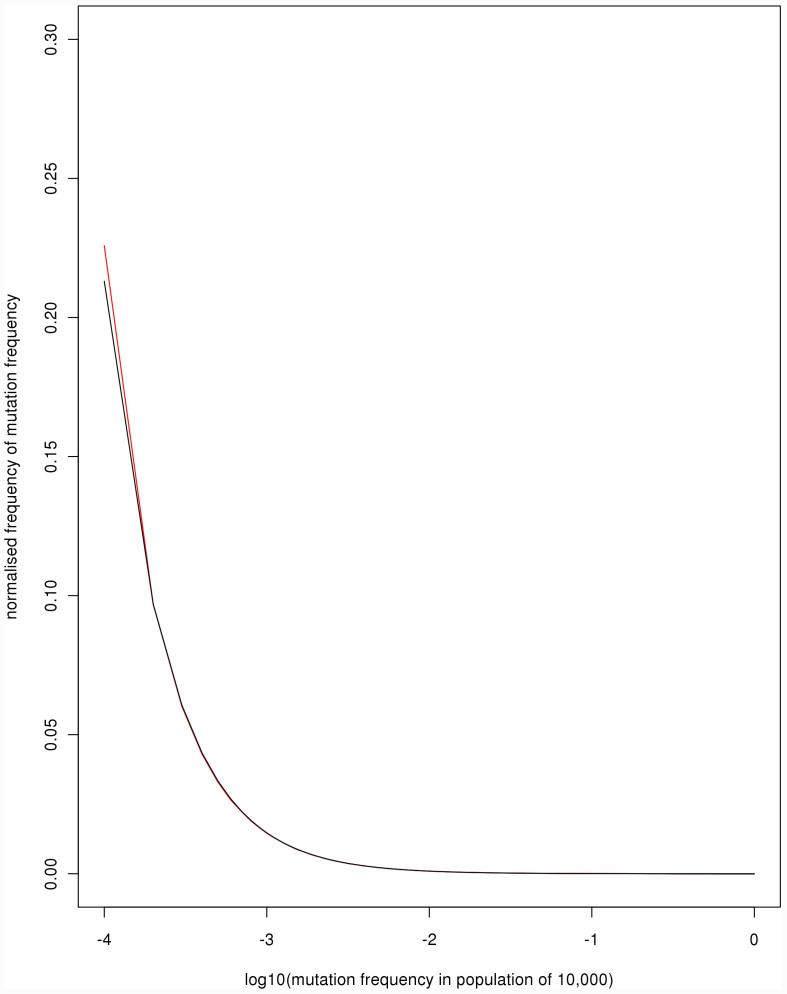
The distribution of mutation frequencies (0.0001–0.9999) according to theory (black) and simulation (red) for 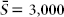. The simulation distribution was created by pooling the frequencies of mutations across models a, c, d, e, f, g and h.

The empirical allele frequency spectrum in the simulations very closely matches the theoretical expectation, with the exception of the lowest frequency, which is slightly higher than expected (Figure 3). This can be explained by the high mutation rate and mode of selection in the Moran model. Low fitness individuals may have new mutations, which will be in the lowest frequency class, that are unlikely to be copied through reproduction. Because selection does not act on the death rate, these individuals will only be removed at a rate proportional to 1/(2*N*) irrespective of their fitness. Thus there is a small accumulation of the lowest frequency class in comparison with the diffusion model.

**Figure 4:**
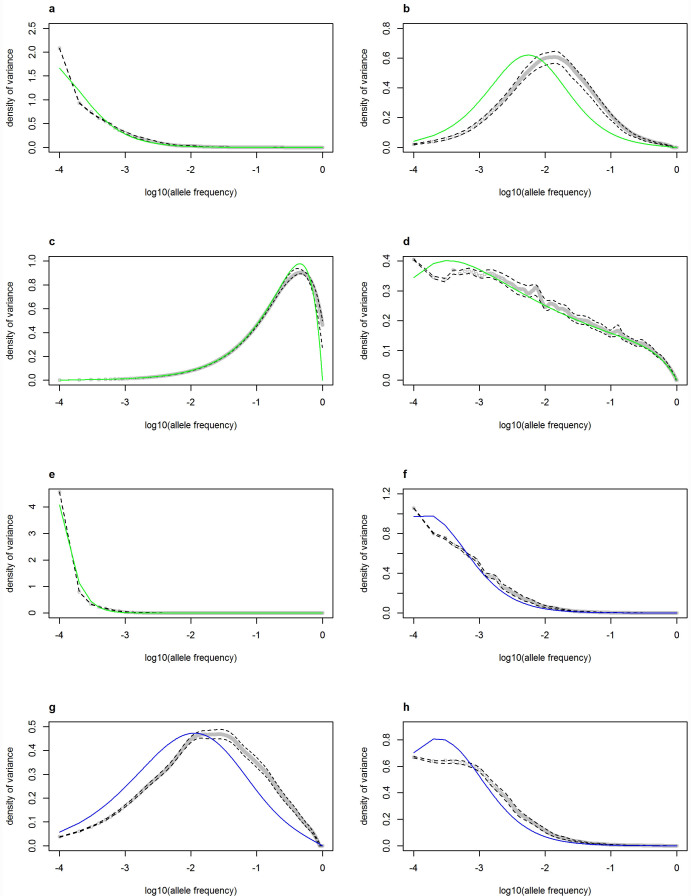
The density of the variance of the trait as a function of allele frequency. The parameters for panels a–h are provided in Table 1 and are (a) 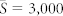, *τ* = 1 (b) 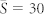, *τ* = 1 (c) 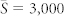, *τ* = 0 (d) 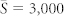, *τ* = 0.5 (e) 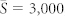, *τ* = 2 (f) 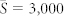, *A* = 10,000,*B* = 10,000 (g) 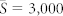, *A* = 4,000,*B* = 40 (h) 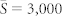, *A* = 4,000,*B* = 4,000. The solid green lines (a–e) show the analytical predictions from the theory in Eyre-Walker^6^, and the blue lines (f–h) are from numerical integration using the saturating function model. The open grey circles give fitted values for each frequency after smoothing using 100 replicate simulations, as described in Methods. The dashed lines give a 95% confidence interval for the fitted values.

The proportion of genetic variance explained by alleles in any frequency class is again broadly similar for simulation and theory (Figure 4). Thus our results support those of Eyre-Walker that, for some parameter combinations, it is possible for very rare alleles to explain a large proportion of the genetic variance in a trait. However two main discrepancies stand out. First, the variance contribution of the lowest frequency class typically appears anomalously larger than expected given the trend in higher frequency classes (Figure 4: models a, d, e, f, h), and larger than the theoretical predictions (Figure 4: models a, d, e, f). This pattern is expected given the observations for the lowest frequency class in Figure 3: there is an excess of new deleterious singletons with large trait effects.

Secondly, ignoring the outlier for the lowest frequency class, there is a general tendency for low frequency alleles to explain a lower proportion of the variance than expected from the theoretical distribution (Figure 4: models a, b, d, e, f, g, h). The only exception to this is model c, where there is no detectable discrepancy between theory and simulations. The strongest discrepancy is for models b and g. Interestingly, although the distributions seem similar, the parameter settings are very different. An additional set of simulations with weaker selection (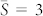, *τ* = 1) in the power-function model also shows an appreciable discrepancy (see Supplementary Figure S17). These discrepancies are likely to be due to interference in the selection of mutations because even though our model assumes a recombination rate between loci of 0.5, selection occurs on whole genomes in the simulation rather than on individual loci as assumed in the diffusion model. As noted earlier, equivalent results would have been obtained if the trait effects were added at the end of the simulations. Thus the effect must be due to a deficit of deleterious mutations in the low frequency classes, and consequent excess of such mutations in the higher frequency classes, even though the overall frequency spectrum closely matches theoretical expectations (Figure 3). Selection is less efficient in removing deleterious mutations, which will also have large trait effects, allowing them to drift to higher frequencies. The reduction in efficiency of selection is in line with earlier results on interference and the Hill-Robertson effect^12^.

We calculated the difference in the proportion of the genetic variance due to mutations with frequency less than or equal to 0.01 between the analytical and empirical results for models a–h using numerical integration over the plotted points in Figure 4 in this frequency interval (between −4 and −2 log_10_ allele frequency). The results are presented in Supplementary Table S2. As expected given Figure 4, models b and g had a 20% and 13% lower proportion in the simulation. The other models had differences between 0% and 2%.

**Figure 5:**
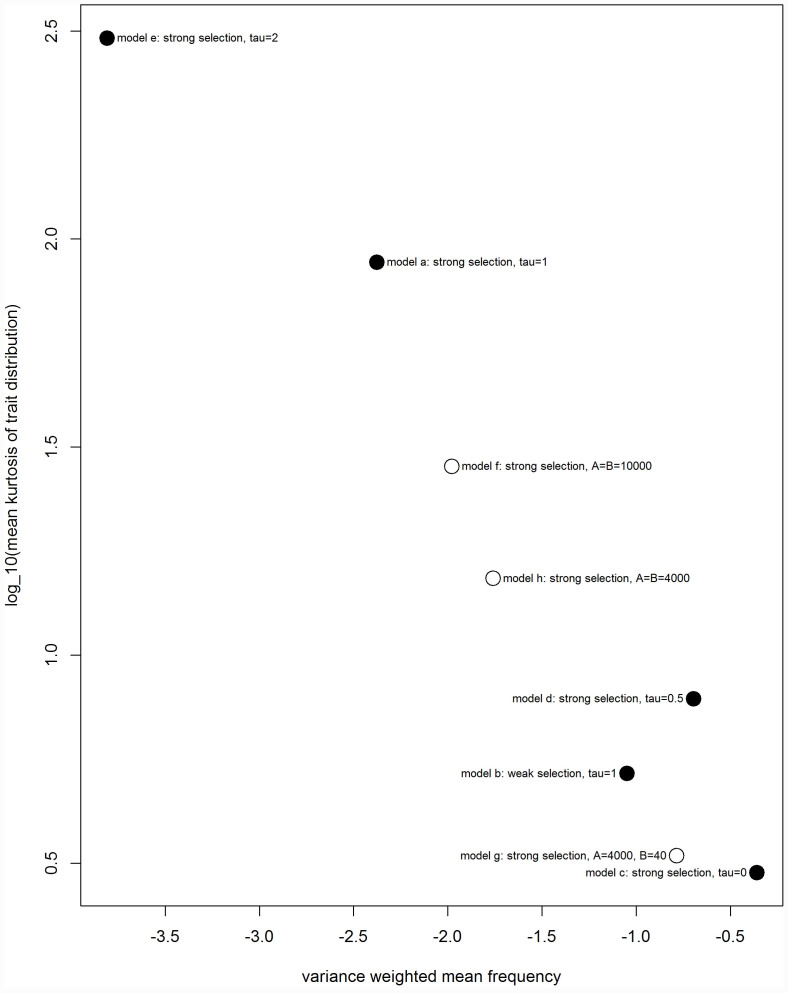
The log_10_ mean kurtosis of the trait distribution computed using the R moments package for each model plotted against the variance weighted mean allele frequency (as displayed in Figure 4). Black points represent the original Eyre-Walker models (a–e) and white points represent the new models (f–h). (Komsta L., Novomesty F. moments: Moments, cumulants, skewness, kurtosis and related tests. R package version 0.13. (2012))

To examine the relationship between the kurtosis of the trait distribution and the extent to which rare alleles were responsible for the variance in the trait (Figure 5), we calculated the mean kurtosis of the trait distribution across the 100 runs of each model and plotted this against the variance weighted mean frequency using the data in Figure 4.

These results show that, for both our models of mutational effects, those scenarios in which rare alleles explain a large proportion of the variance have highly leptokurtic trait distributions, whereas models in which most of the variance is explained by alleles at intermediate frequency are associated with more normal trait distributions (Figure 5). The empirical relationship between the variance-weighted mean frequency and kurtosis does not appear to differ between the saturating function model and the power function model in any consistent way (Figure 5).

## Discussion

Stimulated by the difficulties of explaining an appreciable fraction of complex trait variation despite the availability of extensive data on the genome of individuals, there has been a renewal of interest in evolutionary models of quantitative genetic variation^8^,^9^,^10^,^11^. This follows from earlier developments^4^,^18^, in which much of the analytic theory has been developed. The research focuses on the proportion of genetic variability that can be explained by rare variants, and is significant because, until very recently, the large scale genotyping used in most GWAS studies has involved SNPs that have previously been ascertained in a small panel of individuals, and are necessarily therefore biased towards the effects of common variants.

One aim of this current study has been to compare the analytical predictions of the proportion of variance explained by genes in different frequency classes with results from simulations of a finite population. In general we find that the predictions are close, although there is appreciable discrepancy for some parameter values, which may be explained by Hill-Robertson interference^12^. The second aim has been to investigate the implications of the pleiotropic model for the underlying trait distribution. Here, we observe in our simulations that models in which rare alleles explain a large proportion of the variance give rise to extremely leptokurtic trait distributions, and vice versa. Is the degree of kurtosis implied by the rare alleles model realistic? There appear to be no systematic surveys of the distribution of estimated breeding values, and therefore it is difficult to be sure that the kurtosis recorded in the simulations is extreme. However, on the assumption that most traits do not show extreme kurtosis, and given levels of heritability of 0.5, simple Monte Carlo simulations, assuming Gaussian environmental effects, would suggest that kurtosis in breeding value cannot markedly exceed 10 without notable departure from normality of the trait itself. Taking kurtosis in breeding value of 10 as a somewhat arbitrary cut-off, this would leave models b, c, d, and g as potentially compatible with observations – that is: the weak selection case (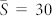), the case with no correlation between trait and fitness (*τ* = 0), and the two low correlation cases (power function model *τ* = 0.5; saturating function model, *B* = 40). Human height, for example, which exhibits high heritability^19^, is not leptokurtic. It is possible, however, that if levels of heritability are less than 0.5, then our argument is not as strong.

It has been noted^6^ that 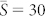 is unrealistically low, given empirical site-frequency spectra. It should also be noted that some of the excess kurtosis in our simulations will be due to the deleterious mutations that contribute to the excess of singletons in the allele frequency spectrum (Figure 3) and also contribute to the excess variance for singletons in Figure 4. This is a feature of the Moran model, and yet is not biologically unrealistic given that individuals with extreme traits may indeed be unlikely to reproduce.

A limitation of the present study is that it does not consider a realistic demography, and although the population size used is approaching current estimates of the human effective population size it does not consider the effect of recent large population size, which will be important in determining the frequencies of recent mutations^20^. These aspects have been recently investigated^10^,^11^. In particular the study of Lohumueller^10^, using Eyre-Walker's model^6^ for mutant effects in a forward-time simulation, finds that when the trait and fitness are partially correlated recent population growth increases the contribution of rare variants to the trait genetic variance. Under all demographic scenarios investigated when *τ* = 0.5 more than half of the additive genetic variance in the trait is caused by very rare alleles (<0.5% allele frequency). When *τ* = 0, he finds that most of the additive genetic variance is caused by common mutations, irrespective of the level of population growth.

Recently it has been shown^9^, using a forward-time simulation package for genome modelling^21^, that, when *τ* = 1 or *τ* = 0, the Eyre-Walker model does not accurately predict results from published studies of Type II diabetes, whereas values of *τ* between 0 and 0.5 cannot be ruled out. This approach points the way forward for using simulation-based analyses to calibrate models of complex trait architecture using published studies. The conclusions of the diabetes study^9^ are consistent with those of the present study which suggests that only values of *τ* of 0.5 or lower give levels of kurtosis in the distribution of phenotypic traits that are close to normal once environmental variation is included.

A further limitation of the present study is that it only explores a fraction of the range of possible joint distributions between mutational effects on fitness and on the trait. Our saturating function model, however, does not appear to produce qualitatively different results from the model of Eyre-Walker. It is likely that the Wishart distribution used by Hill and Keightley^4^ would also give similar results. A limitation of the saturating model is that it assumes an absolute buffering effect of the mutational effect on the trait rather than relative to the trait value of the individual. An improved model would allow for phenotypic or developmental constraint, although implementing this would be a non-additive model. In addition, we have assumed that all fitness effects are deleterious which does not accurately reflect the true distribution^10^. These scenarios are usefully modelled through forward simulations, but do not fit into the theoretical framework of the present study. Furthermore, the assumptions of the underlying modelling approach need further consideration given arguments that pleiotropy is not universal and that most mutations affect a limited number of traits^22^.

Our study confirms that rare variants can explain a large proportion of the genetic variance of complex traits. However, selection on whole genomes within our finite population framework leads to interference effects that lead to a marginal reduction in the variance contributed by rare variants. In addition we note that for many parameter values the pleiotropic model implies a highly leptokurtic trait distribution, and we argue that this imposes an upper limit on the strength of the relationship between trait effects and fitness effects of mutations, in line with recent observations^9^.

## Methods

### Forward-time simulations

The simulations are implemented in C, using the GNU GSL library^23^ for random number generation. A parameter file specifies the haploid population size (2*N*), the length of the simulation, the generation time, the genome mutation rate *U*, the shape parameter, *β*, of the gamma distribution for selection, the *τ* parameter in the conditional distribution for the trait value given by equation (1) (or *A* and *B* for the case of the new equation (2)), the mean strength of selection 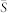, the mean and standard deviation of *ε* (equation (1) and (2)) and the purge interval. The purge interval was used to specify how often we removed fixed mutations from the population to speed up computation time.

We model a population of 10,000 haploid individuals, characterised by a trait value, fitness value and a linked list of mutations which affect the fitness and the trait. The genome of each individual is regarded, for computational convenience, as a single chromosome. We implement an infinite sites model of mutation with free recombination between sites^14^. The population size remains fixed throughout the simulation and each birth event is matched with a death. At the outset, each individual has an arbitrarily chosen trait value of 100 and a fitness value of 1, and carries no mutations. Birth-death events occur according to an exponential random variable with expectation 2 x Generation time/Haploid population size. At each event an individual is chosen uniformly randomly to be replaced. Its genome is replaced by that of two individuals, sampled with replacement from the entire population (including the individual that is replaced) in proportion to their current fitness. Thus it is possible that an individual replaces itself. In copying the genome of the two parental individuals (which might be identical) there is an equal probability that either individual transmits its copy of the allele at each segregating site (i.e. the recombination rate is 0.5). Mutations occur at a rate *U* per genome and only occur at birth. At each birth event the number of mutations is simulated as a Poisson random variable with expectation *U*. The fitness effect, *s*, of a mutation is generated by sampling *S* from the gamma density assumed by Eyre-Walker^6^ (equation (3)) and transforming as *s* = *S*/4*N*.
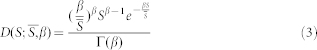


The marginal fitness for that locus is then (1-*s*). Any values of *s* generated that are greater than 1 are assigned the value 1 in the simulation (and the corresponding value of *S* fixed at 4*N*). Conditional on *S*, the trait value is simulated by using either (1) or (2) depending on the model. Trait effects are assumed to be additive across loci. To avoid individuals with negative fitness, fitnesses are assumed to be multiplicative across loci. It should be noted that in the current pleiotropic model there is no need to include trait effects during the running of the simulation, and these could be added afterwards.

### Computing variance contributions

The state of the simulation was outputted at regular intervals in order to assess convergence to equilibrium. The final state of each simulation was combined across 100 replicates, and analysed using R (R Core Team. R: A language and environment for statistical computing. R Foundation for Statistical Computing, Vienna, Austria. (2014)). The aim of the analyses is to compute the contribution by individual loci to the trait variance, which assumes that trait effects are uncorrelated across loci. Loci are not independent due to shared ancestry (with recombination rate 0.5). However, because selection does not act on the trait effects, and because the trait and fitness effects of mutations are uncorrelated, the trait effects are expected to be uncorrelated across loci. However, due to sampling, there will be some residual covariance and rather than ignore this we compute the contribution to the variance by a particular locus as the sum of the column (equivalently row) entries in the trait covariance matrix for this locus. The sum of the elements of the covariance matrix is equal to the total variance in the trait. We show the proportion of the variance in the trait due to covariance terms in Supplementary Table S1.

The variance contributions were summed for all mutations of the same frequency from 1 to 9,999 out of 10,000 individuals. Unobserved frequencies had zero variance contribution. The variance contribution for each allele frequency (from 0.0001 to 0.9999) was then averaged across the 100 runs (and hence any frequency class that was never observed across the 100 runs had zero variance contribution). Some variance contributions close to zero were negative because of the inclusion of covariance terms.

The averaged variance contributions for each allele frequency class were smoothed within models using the R lokern package to remove noise (Herrmann E., Maechler M. lokern: Kernel Regression Smoothing with Local or Global Plug-in Bandwidth. R package version 1.1–5. (2013)). The smoothing was performed after log_10_ transformation of the allele frequencies. The fit was obtained, using the function glkerns(), by supplying an estimate of the variance of the mean variance contribution for each allele frequency, *x*. This estimate was obtained by computing the observed variance of the variance contribution among the 100 replicates, and then dividing by 100 to obtain an estimate of the variance of the mean. A smoothed estimate of this variance was then made by using glkerns() with default parameter settings. To do this the square root of this variance (i.e. the standard error) was supplied, and then the smoothed value was squared. This was supplied to glkerns(), along with the mean variance contribution for each *x*. We specified that the values of *x* were designed rather than random, and chose defaults for all other parameters. The predicted values were multiplied by the coefficient log_e_(10)*x*, where *x* is the allele frequency. At this stage all estimated variance contributions <0 were considered negligible and set equal to 0. This function was then converted to a density by computing an approximate normalising constant using the trapezoidal rule. This can then be compared with the analytical solution obtained by Eyre-Walker^6^, expressed as V(*x*)/V_T_, the variance contributed by all mutations of frequency *x* divided by the total variance in the trait. Bootstrap estimates were computed by simulating 2,000 new data sets obtained by adding re-sampled residuals to the fitted values of the initial fit^24^. To account for non-constant variance the 50 closest residuals were chosen for each point. Detailed genetic variance plots showing the creation of Figure 4 are provided in the Supplement.

To account for a finite population size we modified the calculation of Eyre-Walker (equation 7 in the original paper^6^), using the Mathematica notebook kindly supplied by the author, to have integration limits from 0.0001 to 0.9999 so that the analytical solution could be compared with the simulation results (Wolfram Research, Inc., Mathematica, Version 8.0, Champaign, IL (2010)). The updated expression for V(*x*)/V_T_, the density of the variance, is thus
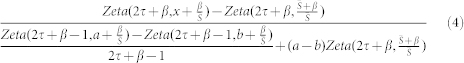
where *x* is the allele frequency and Zeta is the Hurwitz Zeta function, with integration limits of a = 0.0001 and b = 0.9999. We then approximated the Hurwitz Zeta function in R and superimposed the analytical solution into the plot with a coefficient of *x*.log_e_(10) to achieve a change of variables to the log_10_ allele frequency scale. Before plotting we added an additional numerical normalisation. (With the change of variables to a log scale there is necessarily some granularity in the estimation of the density at the left hand end of the plots because this is evaluated only at the points (1/10000), (2/10000),… etc. Thus even if the relative frequency evaluated at these points is directly proportional to the diffusion density there is potential discrepancy when plotting the theoretical density with that evaluated from the simulations, arising from the approximate nature of the trapezoidal integration used to scale the simulated points. In order to minimise this, to ensure that the theoretical and simulation results are compared on the same scale, we have therefore adjusted the diffusion prediction by also using trapezoidal integration based on the same evaluation points. In this case, for most models, the integration constant for the theoretical density deviates from 1 by <0.001, and is undetectable in the figures, but for model e, because most of the density is concentrated towards zero, there is appreciable discrepancy of around 0.08.)

The approach taken for comparing the discrete simulation results with the continuous analytical solution^6^ can be justified as follows. We equate the sample average variance contributed by all mutations of frequency x in our simulations, T(*x*), with the expected variance contribution in the interval (*x*,*x* + Δ*x*), approximated by Δ*x*V(*x*), where V(*x*) is the diffusion solution^6^ and Δ*x* = 1/(2*N* − 1). In the limit as *N* → 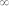, T(*x*) → 0, and (2*N* − 1)T(*x*) → V(*x*) in expectation. Our normalised function estimates V(*x*)/V_T_, where V_T_ = ∫V(*x*), because it estimates 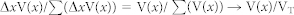 as *N* → 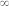.

The variance contribution under the saturating function model (equation (2)) was obtained numerically in Mathematica. In this case we substitute equation (2) for equation (1) and evaluate the integral in equation 3 of Eyre-Walker, by integrating over *ε* analytically and integrating over *S* numerically using the NIntegrate function. This was then subject to the same change of variables approach, and normalisation using trapezoidal integration, for comparable plotting with the empirical data.

To obtain the site frequency spectrum shown in Figure 3, we solved the following analytically using Mathematica
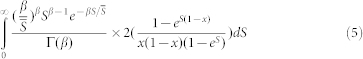


The left hand side of expression (5) is equation 2 of Eyre-Walker^6^ and the right hand side is equation 4 of Eyre-Walker^6^. It is the expected passage of time for mutations at frequency *x* with selective strength *S*.

## Author Contributions

T.L.N. and M.A.B. devised the study together, and contributed to the coding and analyses of the results, and wrote the manuscript together.

## Additional Information

**Accession codes:** Relevant code used in preparing this manuscript can be accessed at https://github.com/terinorth/.

## Supplementary Material

Supplementary Information Supplementary Information

## Figures and Tables

**Table 1:** Parameters of models implemented

Model	Parameter combinations	Comment
a	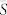 = 3,000, τ = 1	Original EW model
		Strong selection
		Absolute trait effect and fitness effect linearly correlated
b	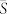 = 30, τ = 1	Original EW model
		Weak selection
		Absolute trait effect and fitness effect linearly correlated
c	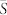 = 3,000, τ = 0	Original EW model
		Strong selection
		Absolute trait effect and fitness effect uncorrelated
d	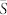 = 3,000, τ = 0.5	Original EW model
		Strong selection
		Absolute trait effect and fitness effect weakly correlated
e	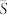 = 3,000, τ = 2	Original EW model
		Strong selection
		Quadratic relationship between absolute trait effect and fitness effect
f	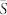 = 3,000,A = 10,000,B = 10,000	Saturating function
		Strong selection
		Absolute trait effect threshold of 10,000 before noise term (1 + ε)
		Linear relationship between trait and fitness near origin with gradient 1 before noise term δ(1 + ε)
g	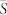 = 3,000,A = 4,000,B = 40	Saturating function
		Strong selection
		Absolute trait effect threshold of 4,000 before noise term (1 + ε)
		Linear relationship between trait and fitness near origin with gradient 100 before noise term δ(1 + ε)
h	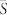 = 3,000,A = 4,000,B = 4,000	Saturating function
		Strong selection
		Absolute trait effect threshold of 4,000 before noise term (1 + ε)
		Linear relationship between trait and fitness near origin with gradient 1 before noise term δ(1 + ε)
