# Supplementary material for: Complex trait architecture: the pleiotropic model revisited
Source: Sci Rep. 2015 Mar 20;5:9351. doi: 10.1038/srep09351 (PMC4366851; doi:10.1038/srep09351)
Supplement: Supplementary Information [file srep09351-s1.pdf]

Complex trait architecture: the pleiotropic model revisited

North T-L and Beaumont MA

SUPPLEMENTARY INFORMATION

Detailed genetic variance plots

Note that panel letters in these supplementary figures **do not refer to models**. The models are clearly referenced in the headings.

The detailed genetic variance plots show, for each model a-h, the process of deriving the figures presented in the main paper Figure 4. In addition, we present plots of the theoretical and empirical variance contributions across frequencies.

Panel a in the first plot for each model shows the empirical mean variance contribution as plotted points with a smoother derived using the R lokern package[1] as a red solid line. A 95% confidence interval for the smoother derived as described in the Methods is represented by two dashed red lines.

Panel b in the first plot for each model displays the smoother and confidence interval after a change of variables by multiplying the values by $\log_e(10) \times 10^y$ where $y=\log_{10}(x)$ and x is the allele frequency. This approach, as taken by Eyre-Walker[2], ensures that the area under the curve is proportional to the probability.

Panel c in the first plot for each model has been normalised to ensure that the area under the curve is 1. The green and blue lines represent the analytical result with altered integration limits (see Methods) for comparison. This was calculated using Mathematica[3] (green lines) or numerical integration (blue lines). This is the same plot as presented in Figure 4. The values from the lokern smoother are plotted as points rather than a solid line as in panel b.

Panel d in the first plot for each model plots the residuals from the glkerns smoother[1] against the $\log_{10}$ allele frequency.

The second plot for each model shows the theoretical variance contribution for each allele frequency plotted against the raw empirical mean variance values following a transformation of the empirical (upper panel a) or the theoretical (lower panel b) results to ensure a linear expected relationship.

Supplementary Figure S1: Model a

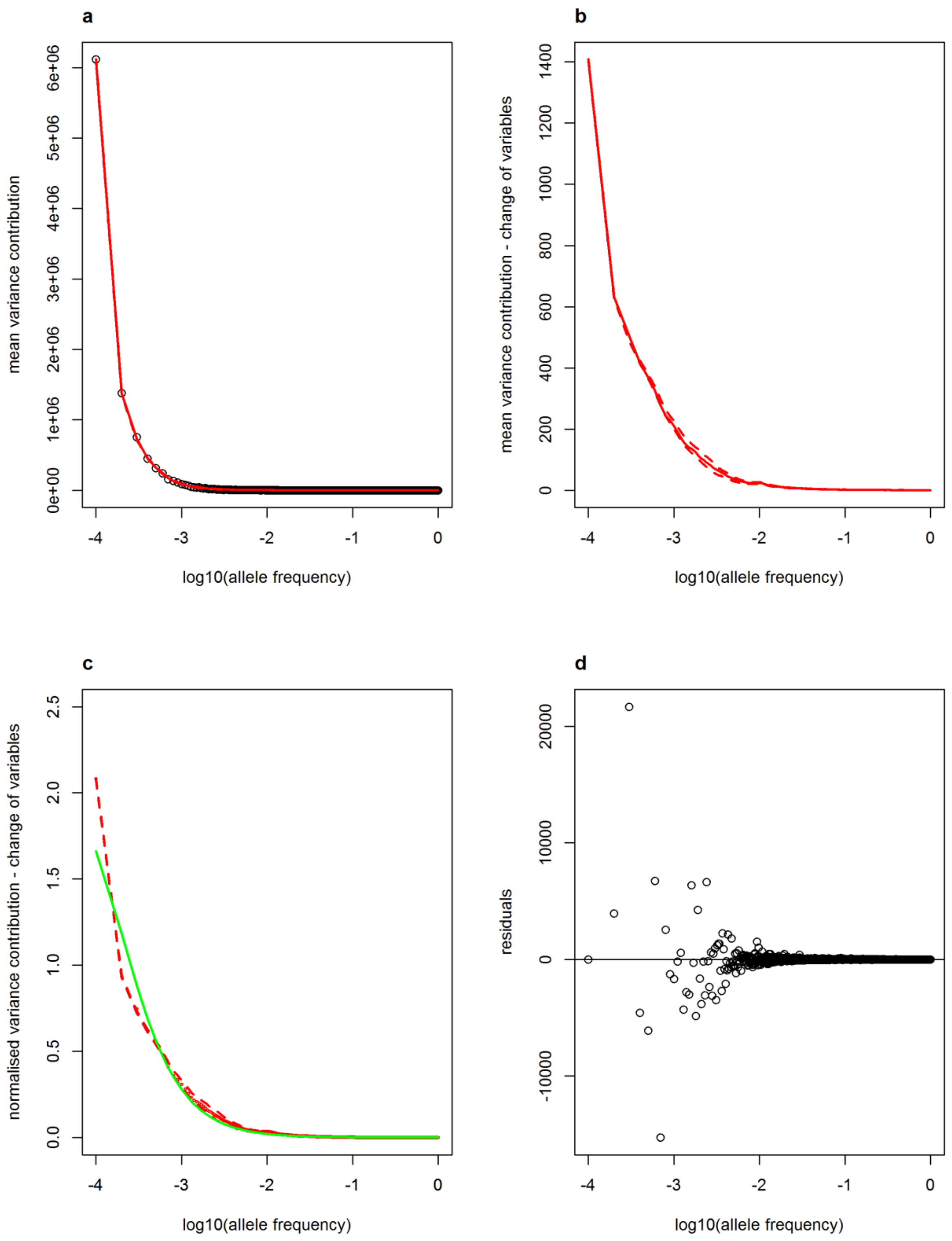

Supplementary Figure S2: Model a

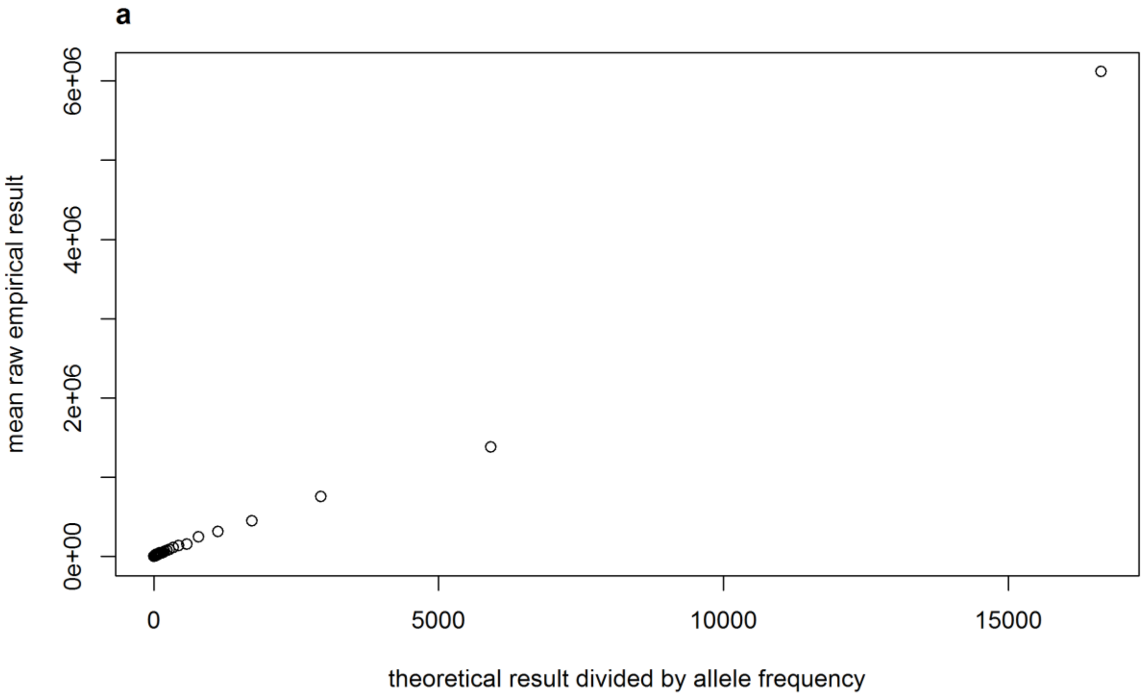

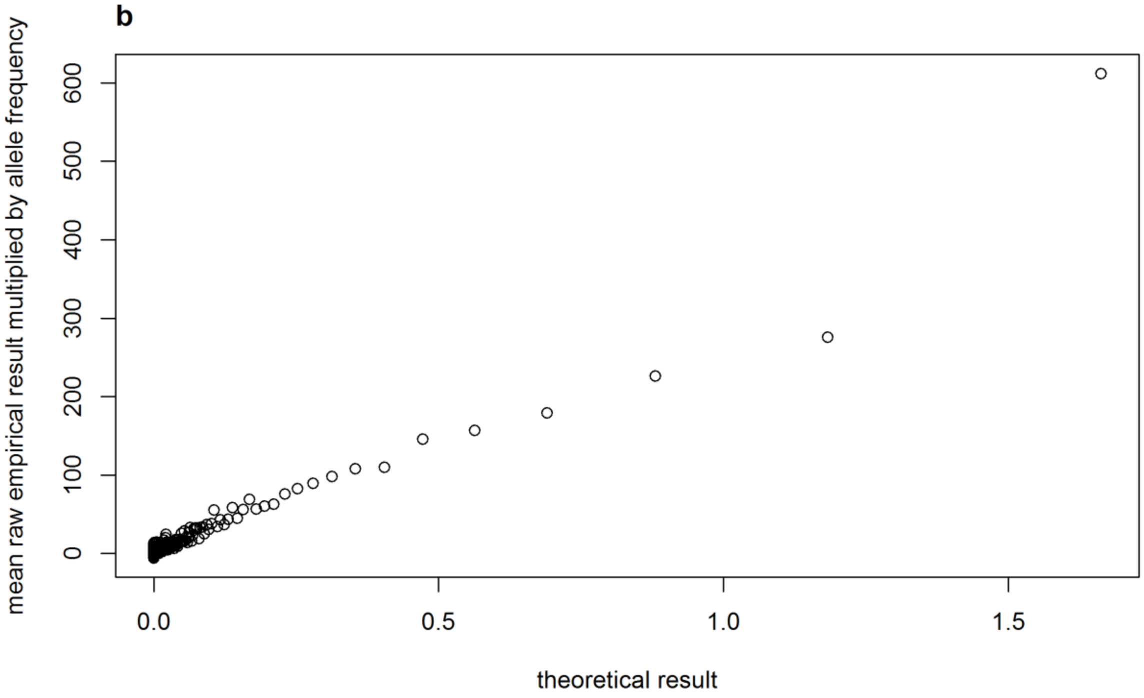

Supplementary Figure S3: Model b

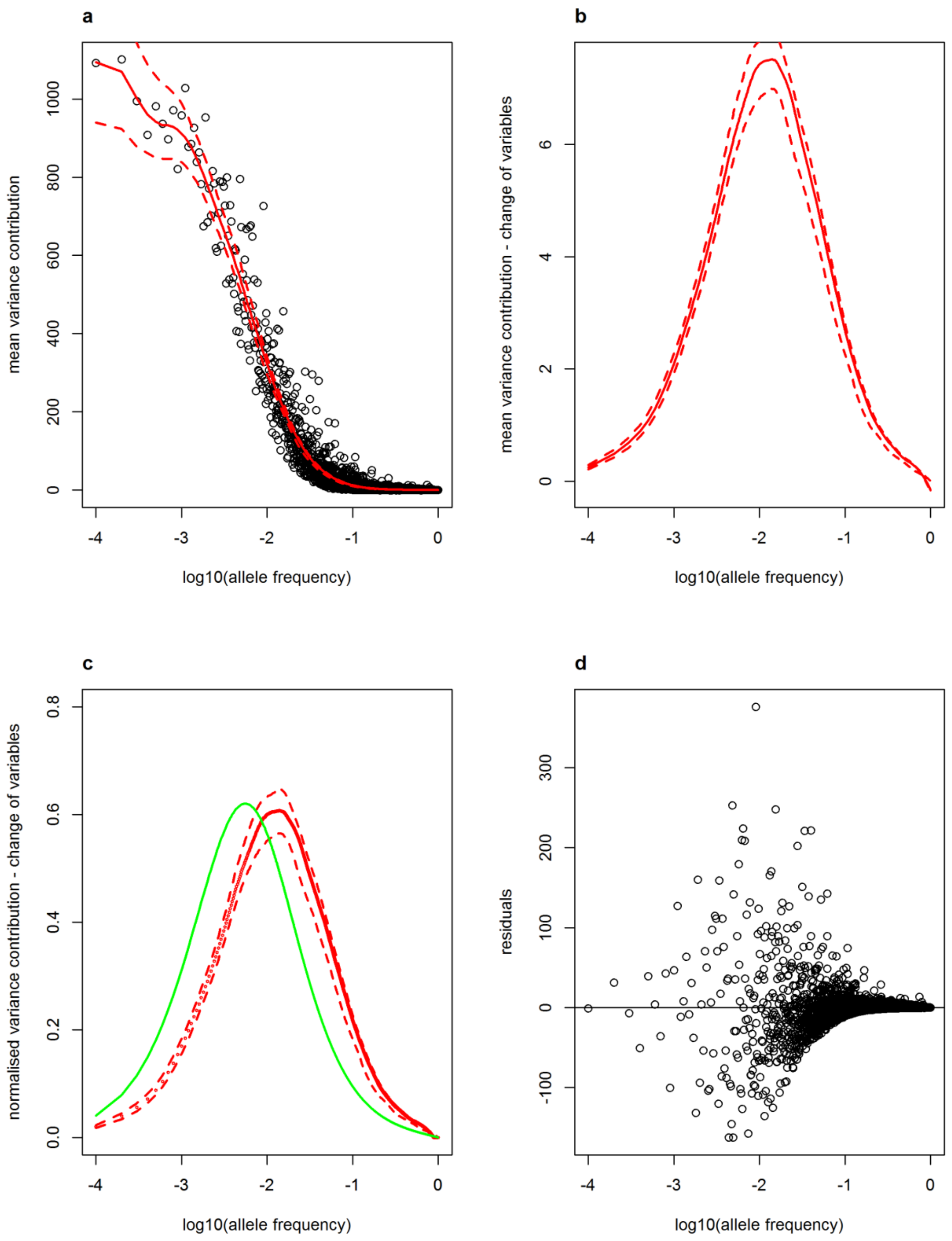

Supplementary Figure S4: Model b

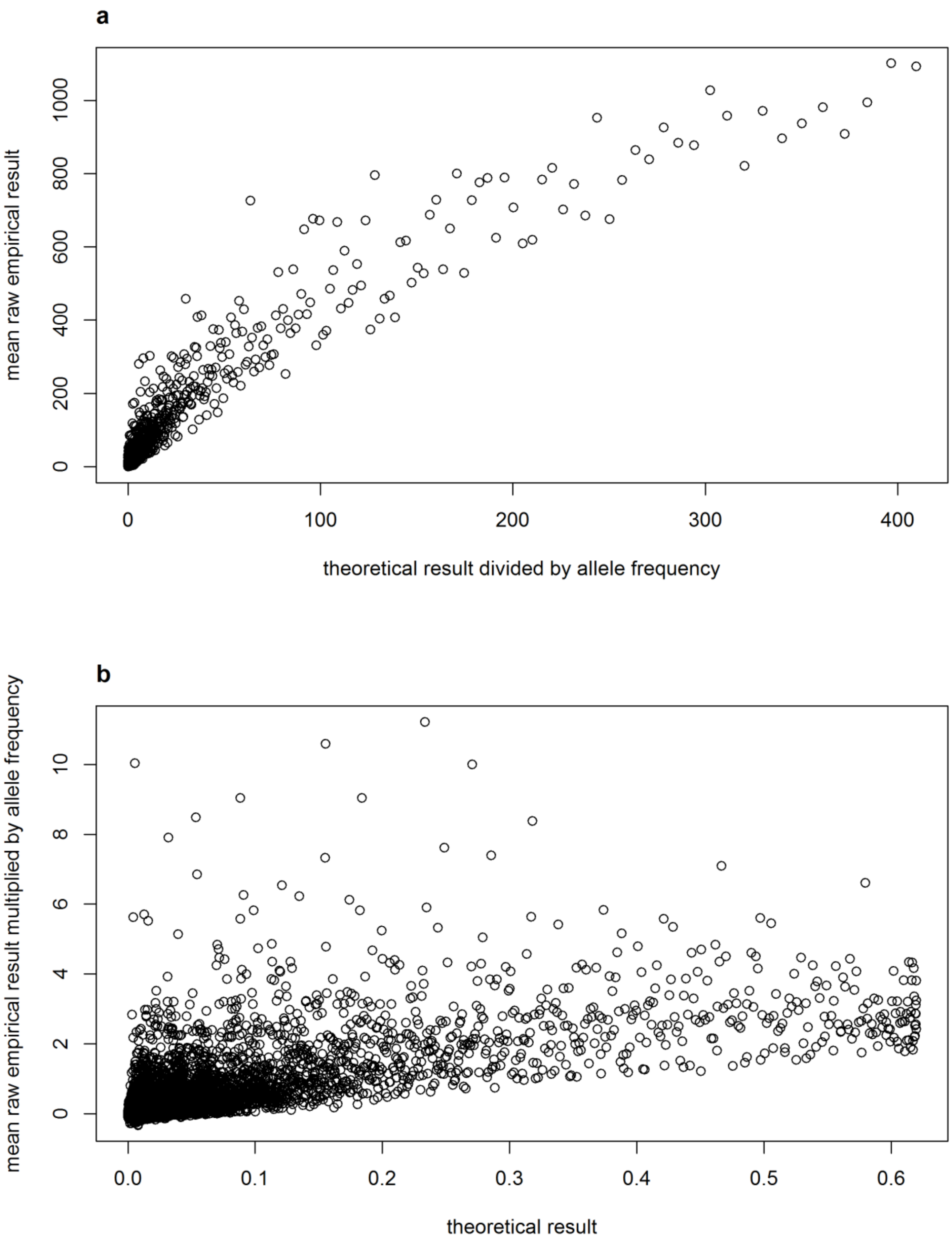

Supplementary Figure S5: Model c

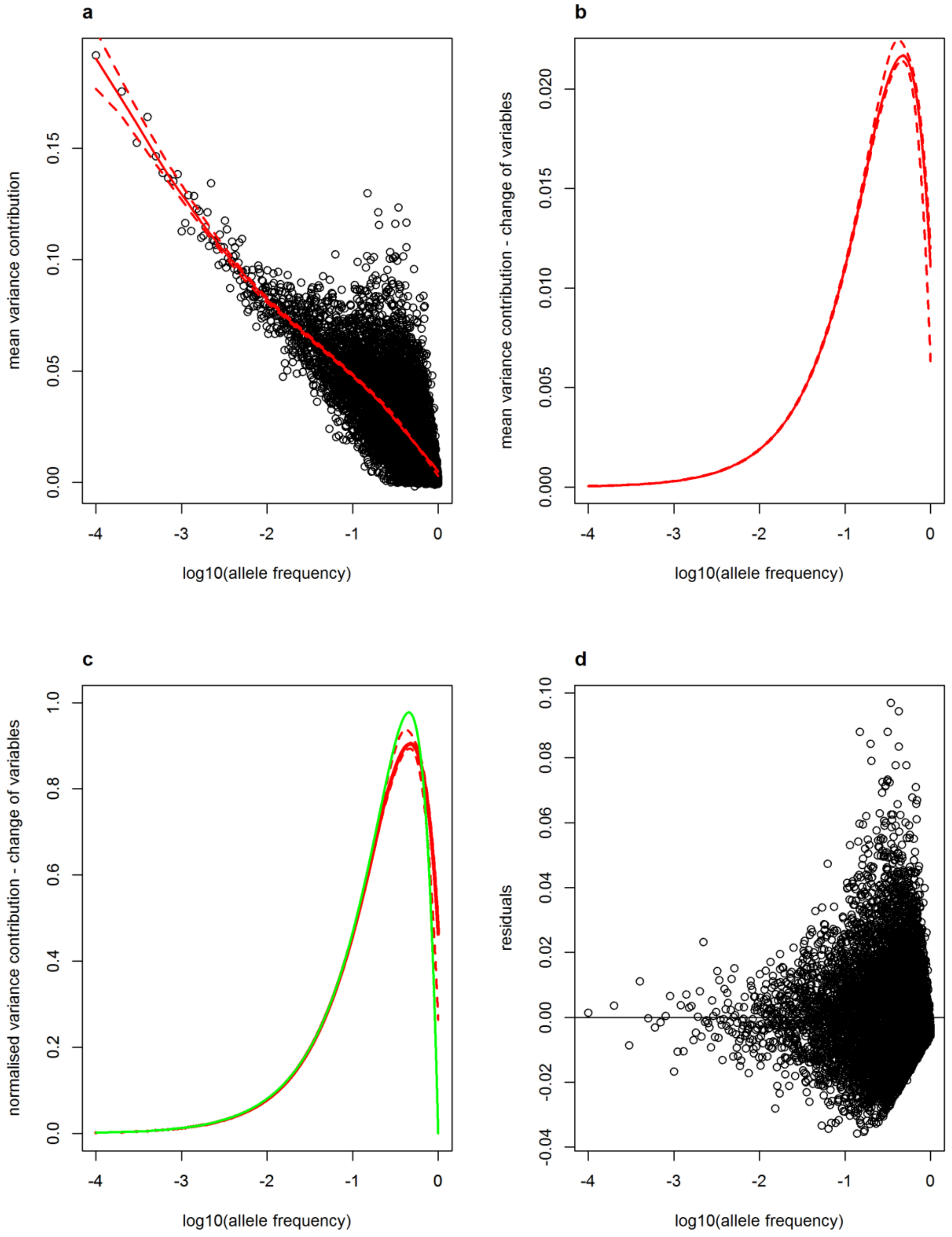

Supplementary Figure S6: Model c

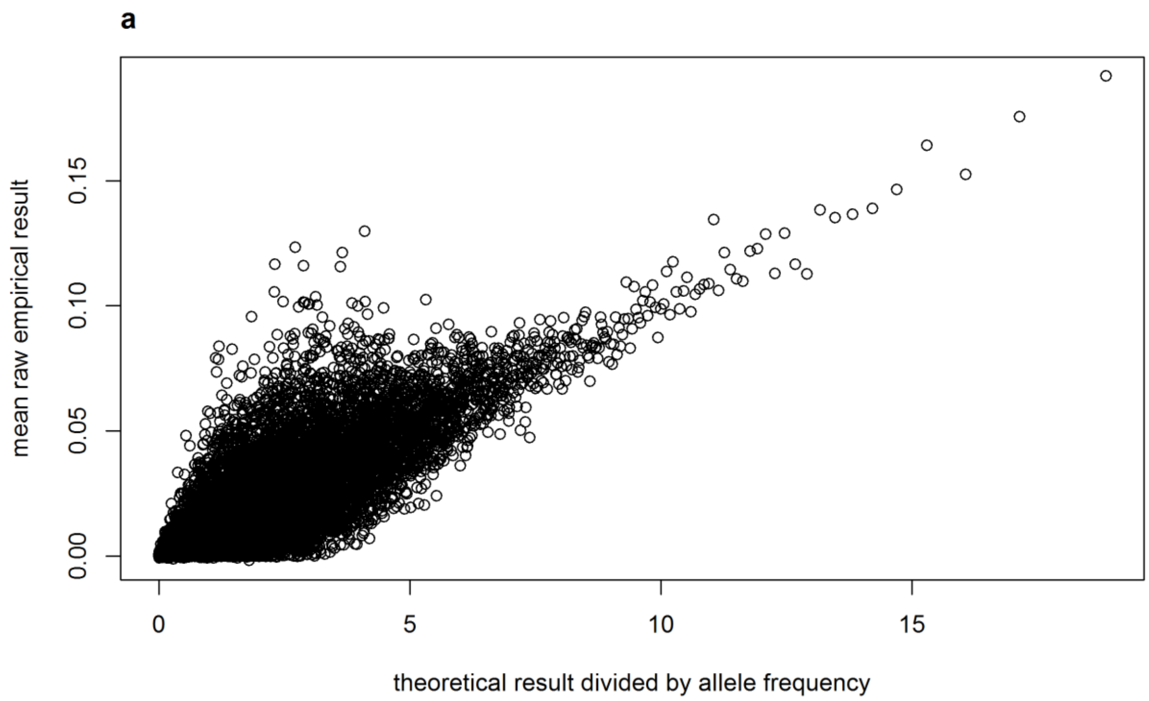

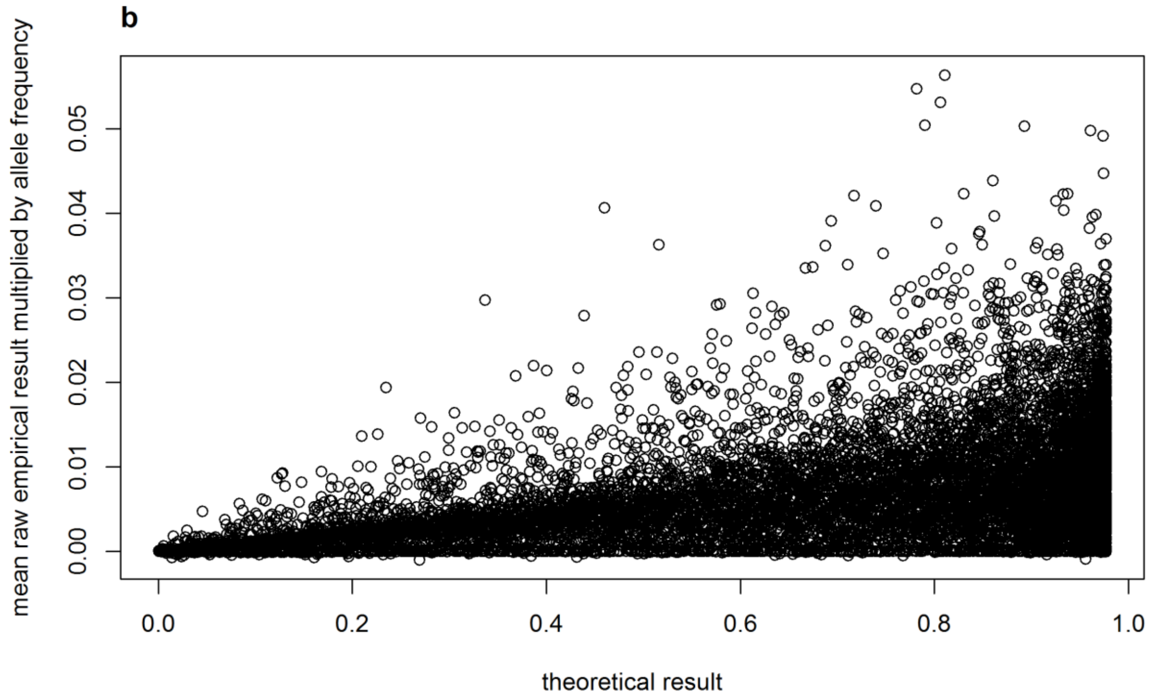

Supplementary Figure S7: Model d

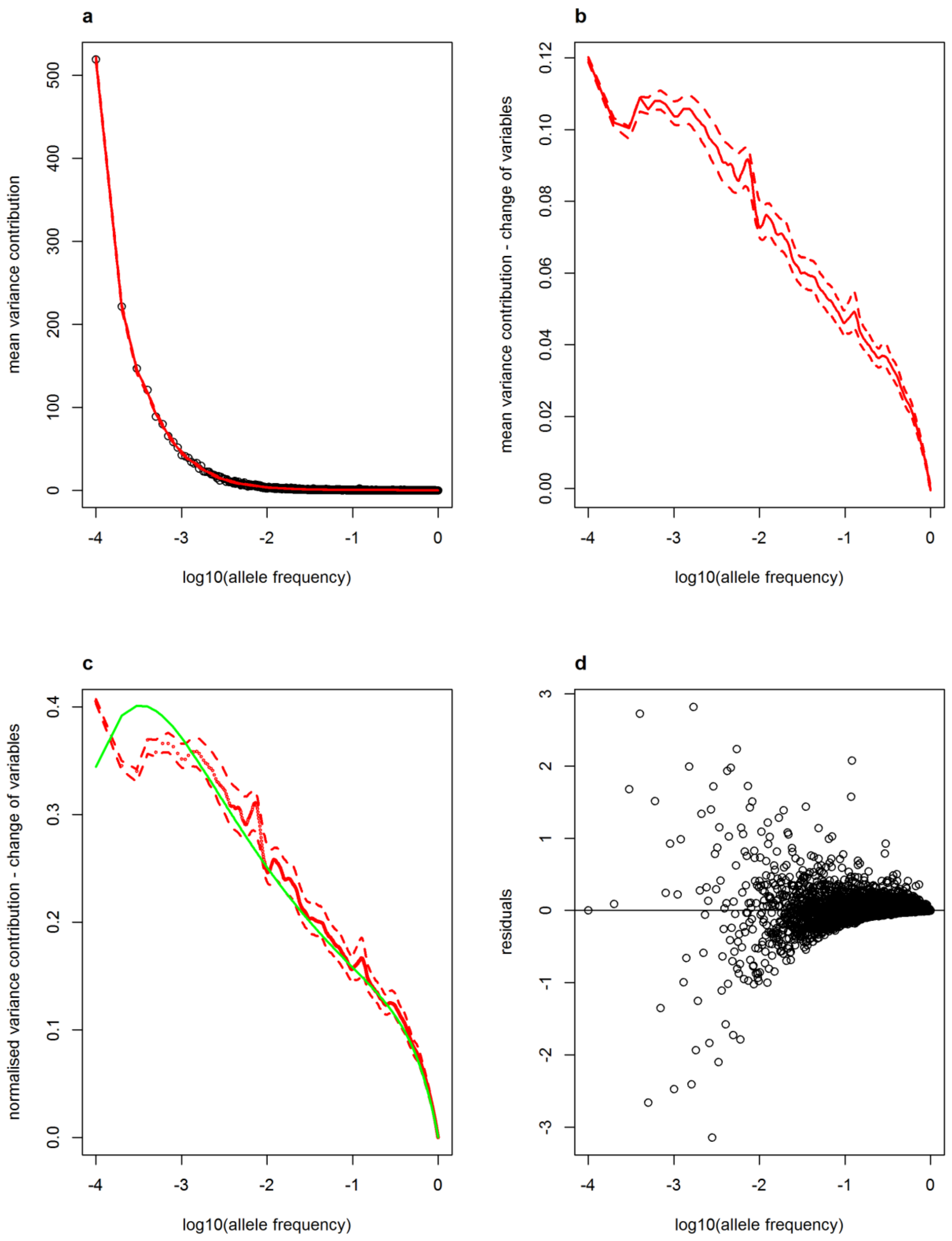

Supplementary Figure S8: Model d

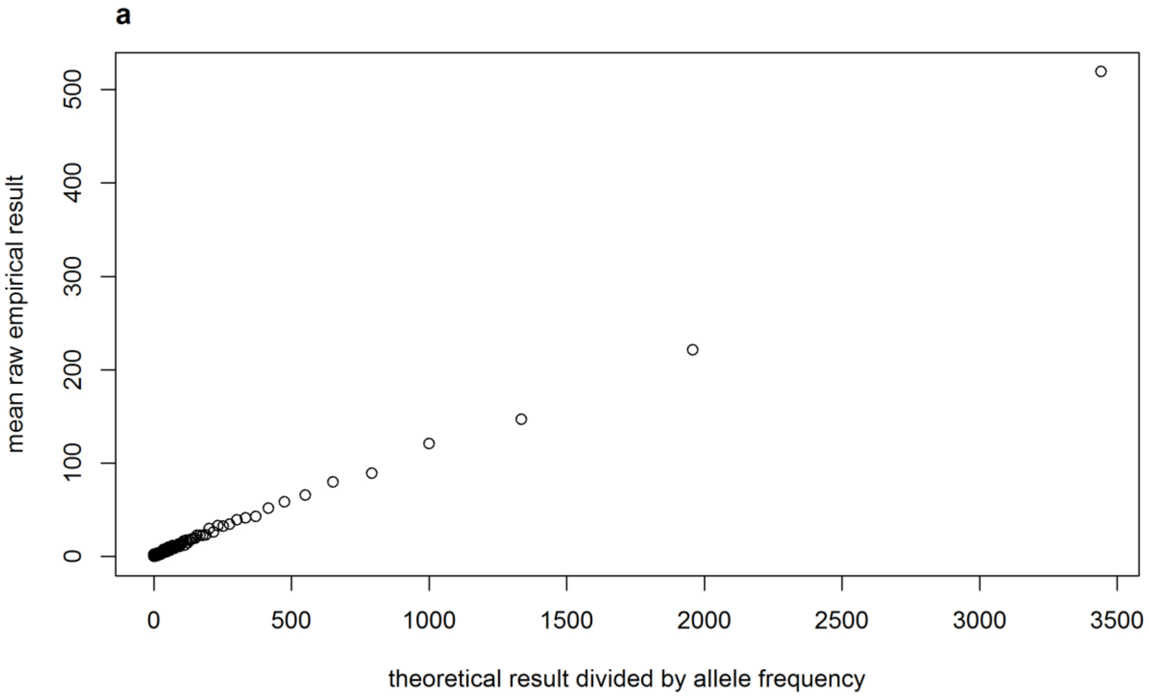

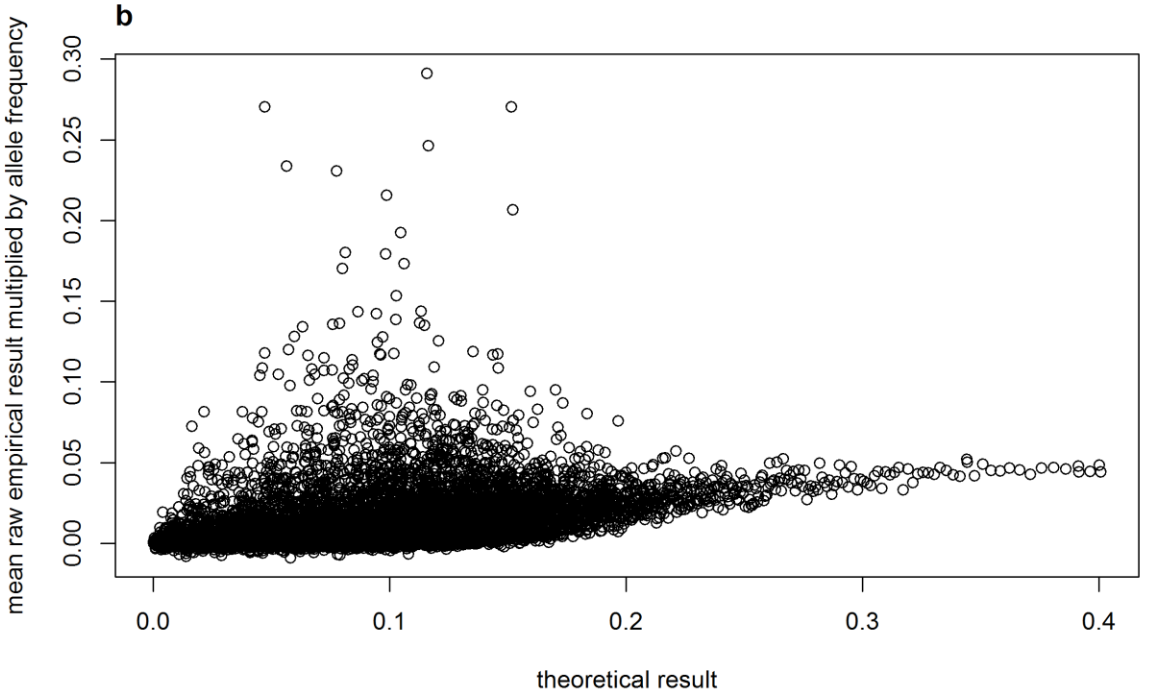

Supplementary Figure S9: Model e

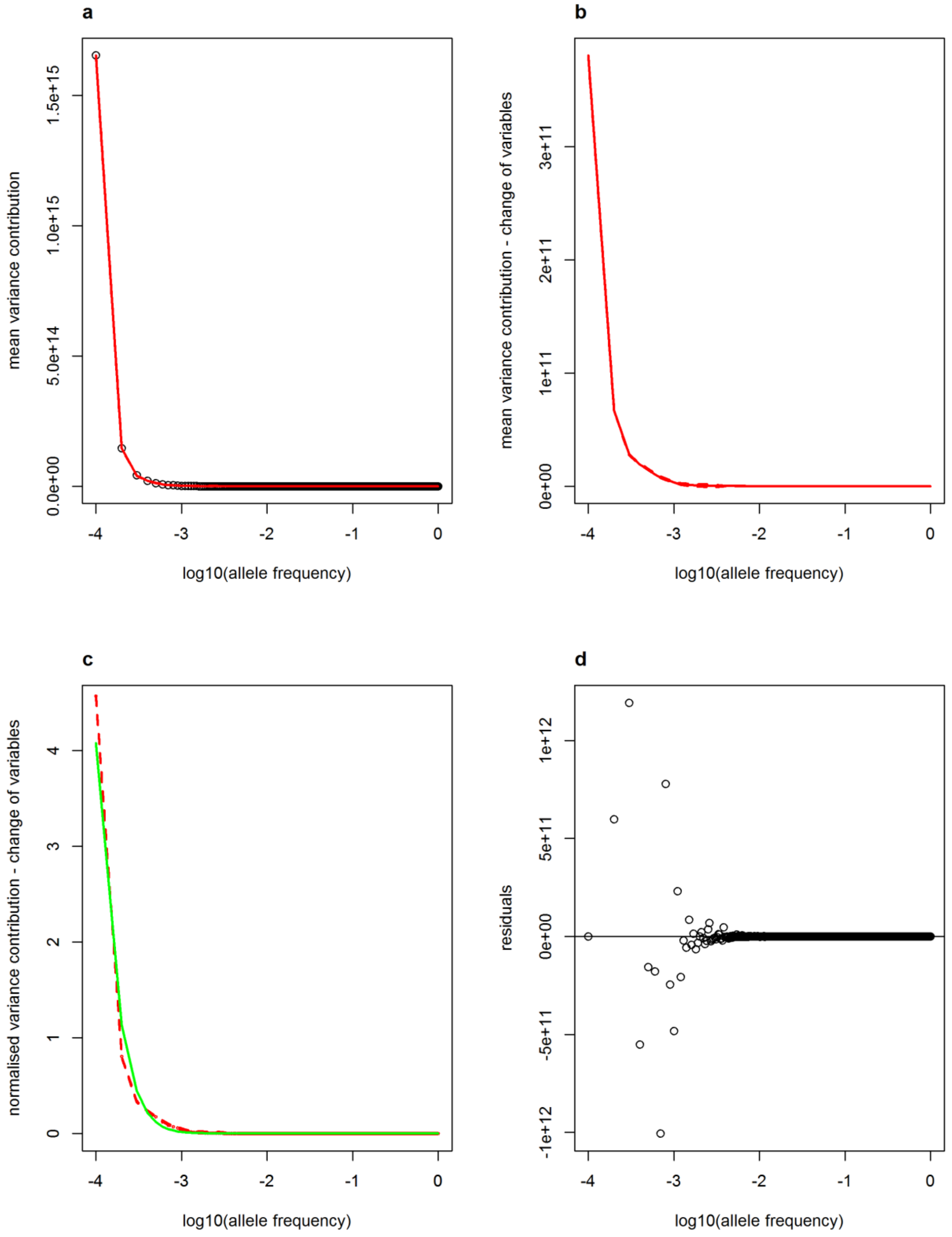

Supplementary Figure S10: Model e

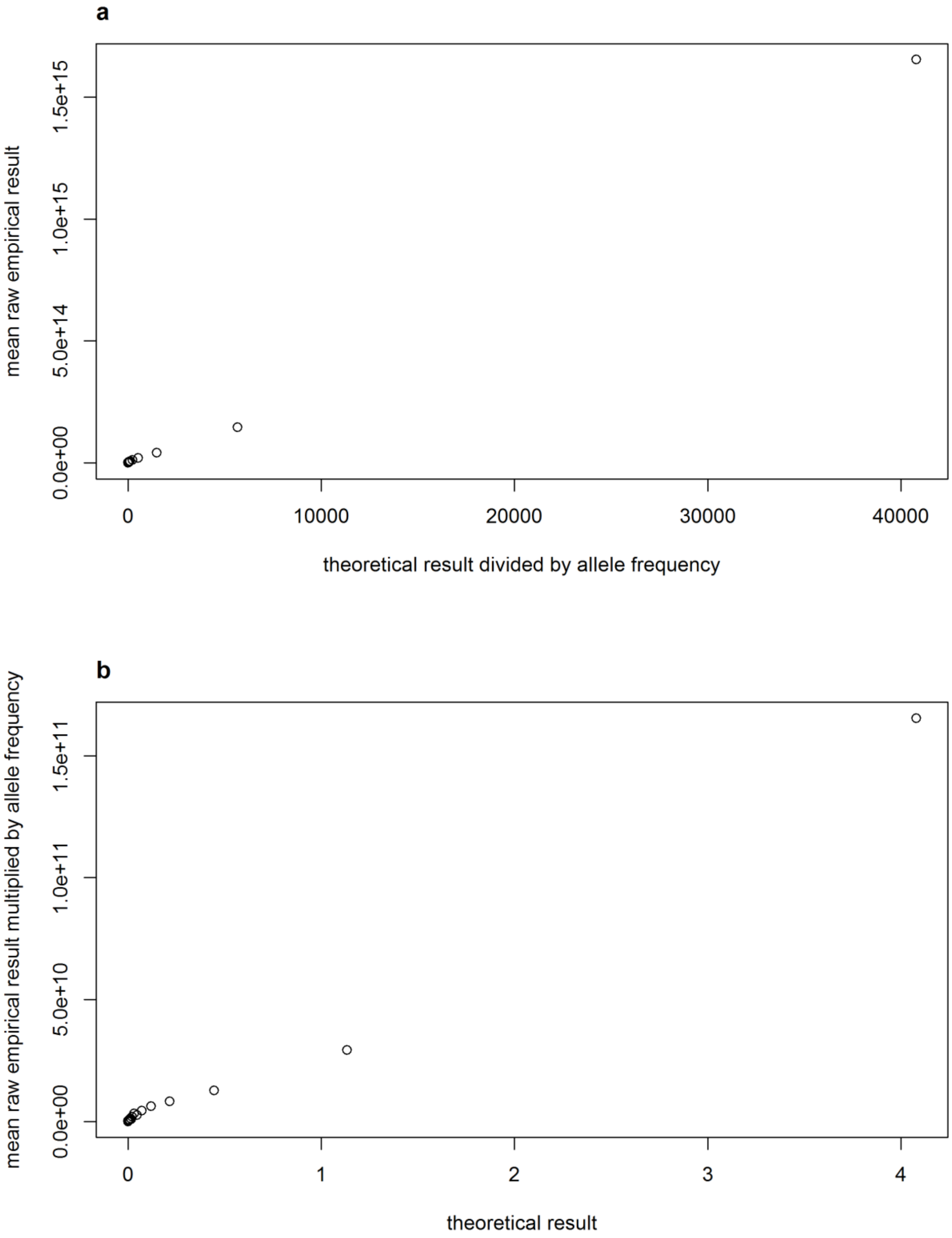

Supplementary Figure S11: Model f

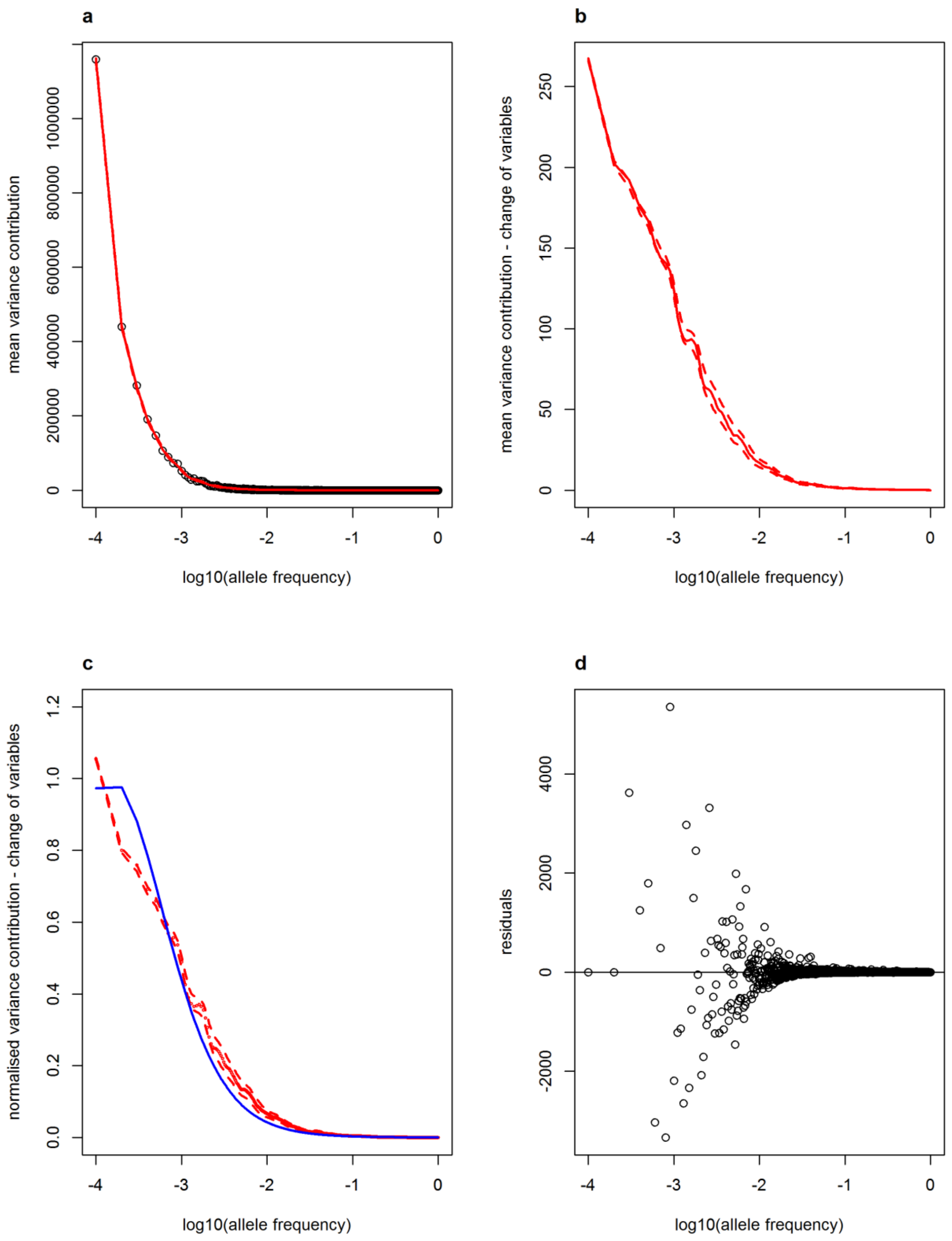

Supplementary Figure S12: Model f

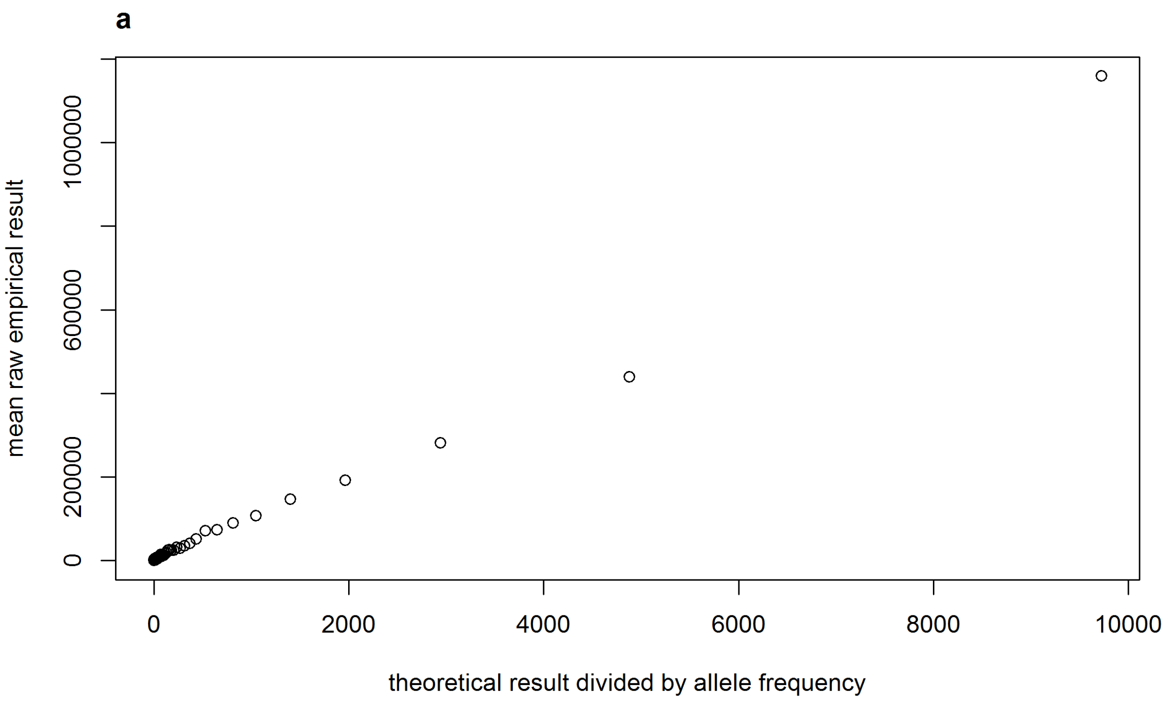

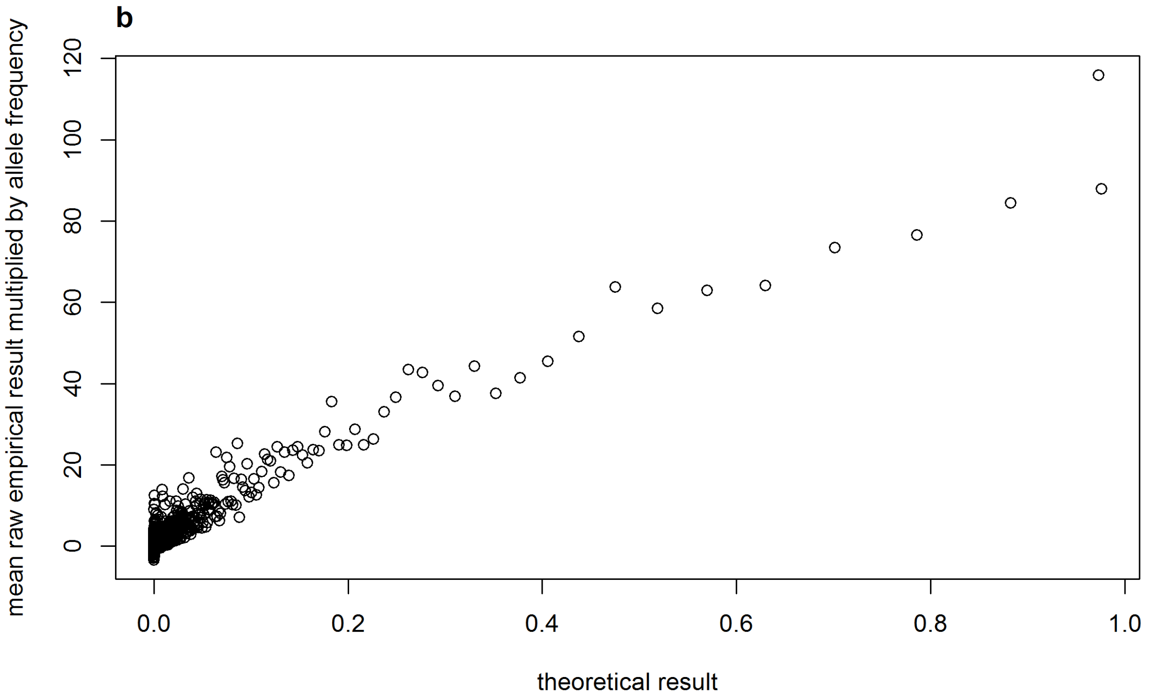

Supplementary Figure S13: Model g

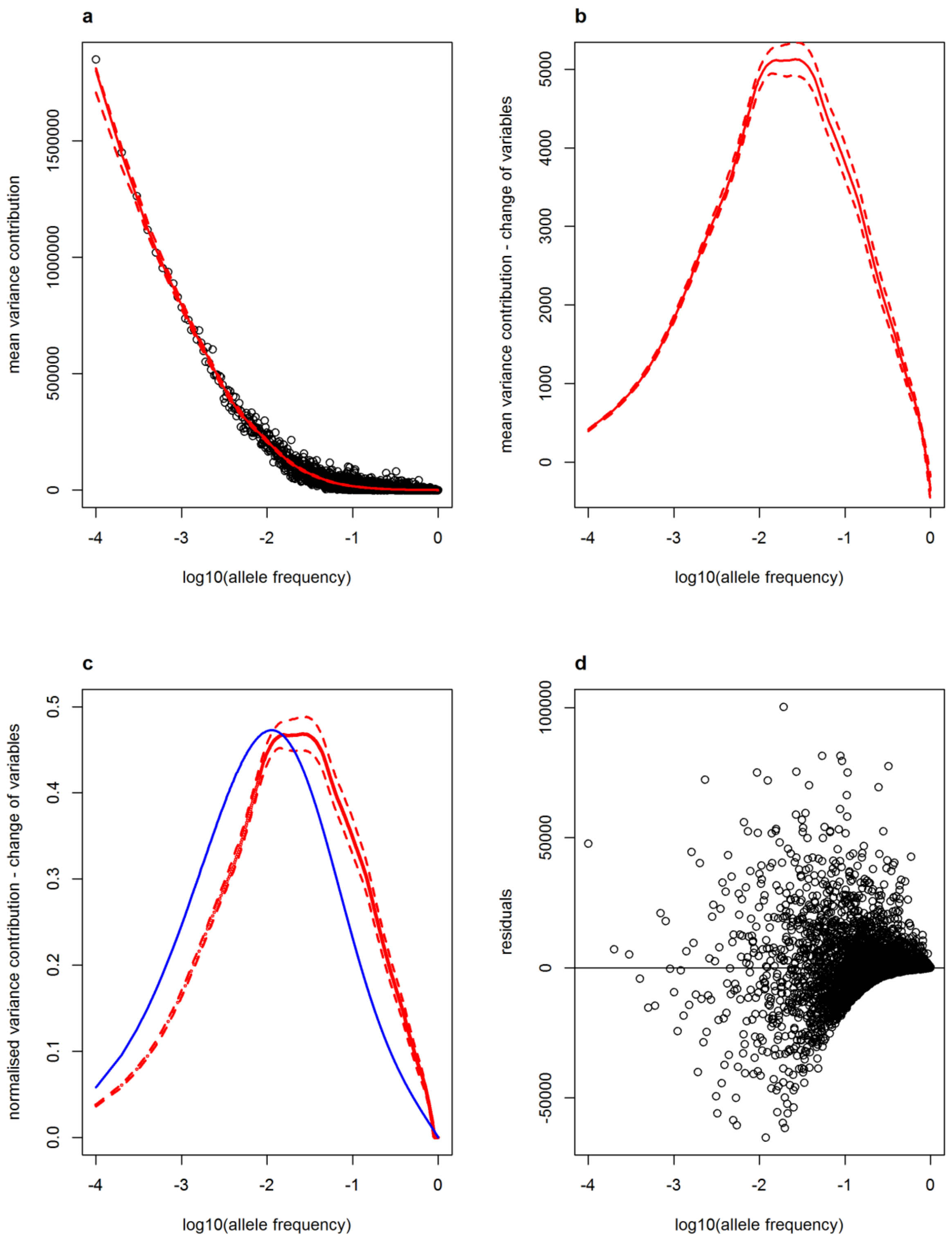

Supplementary Figure S14: Model g

a

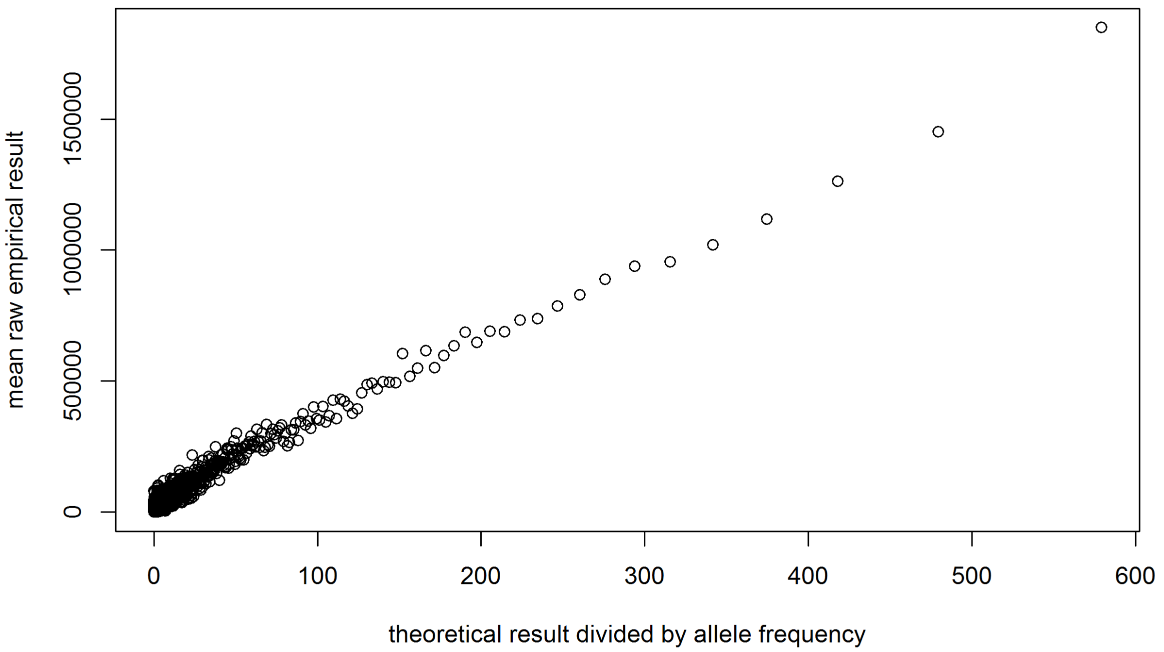

b

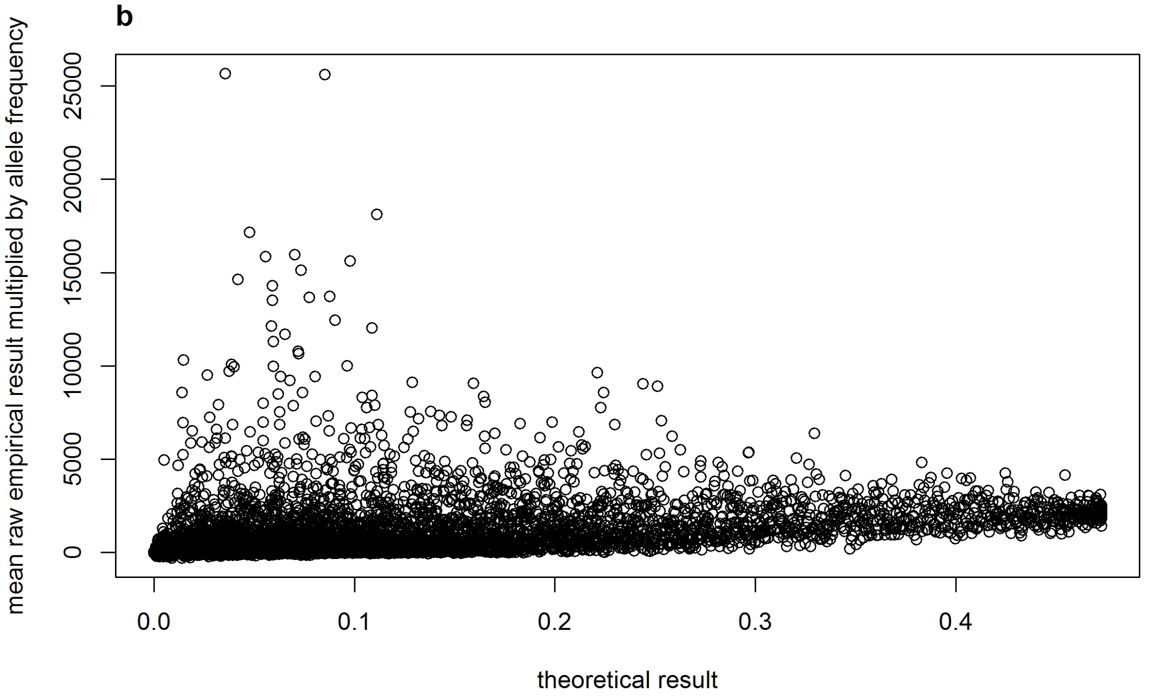

Supplementary Figure S15: Model h

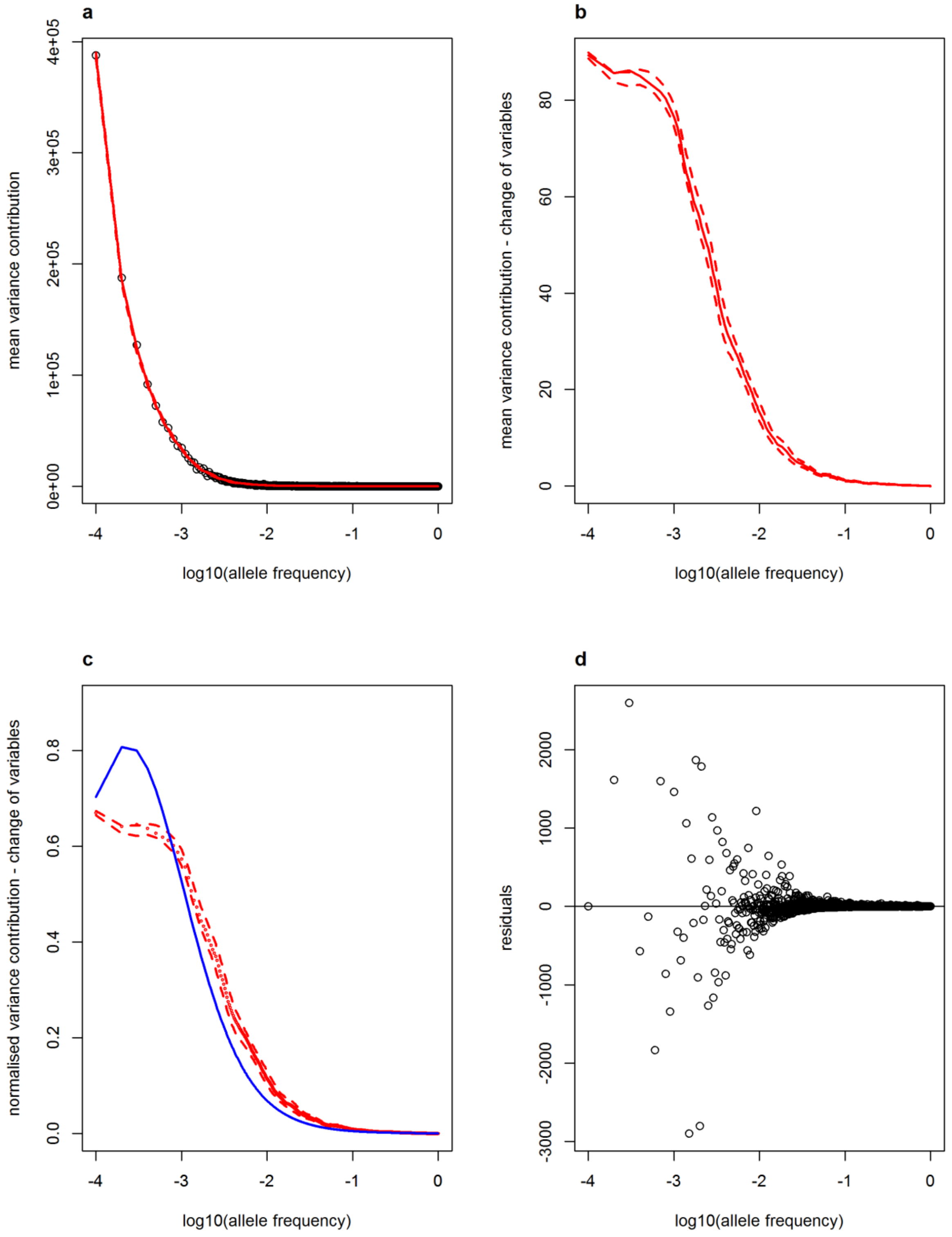

Supplementary Figure S16: Model h

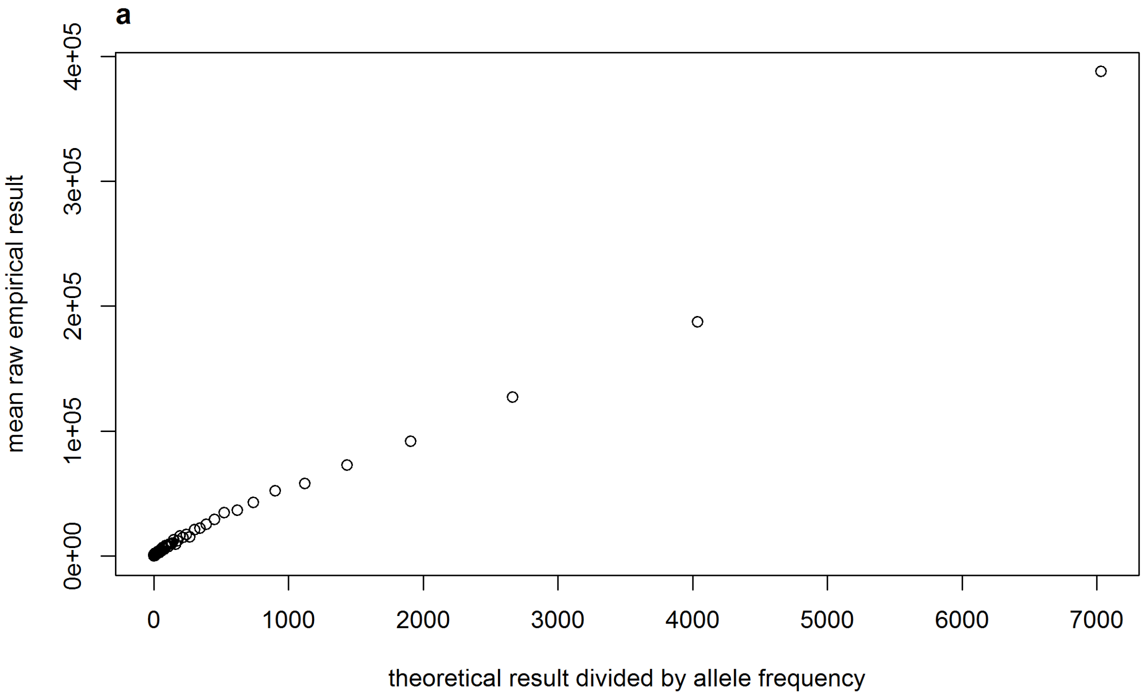

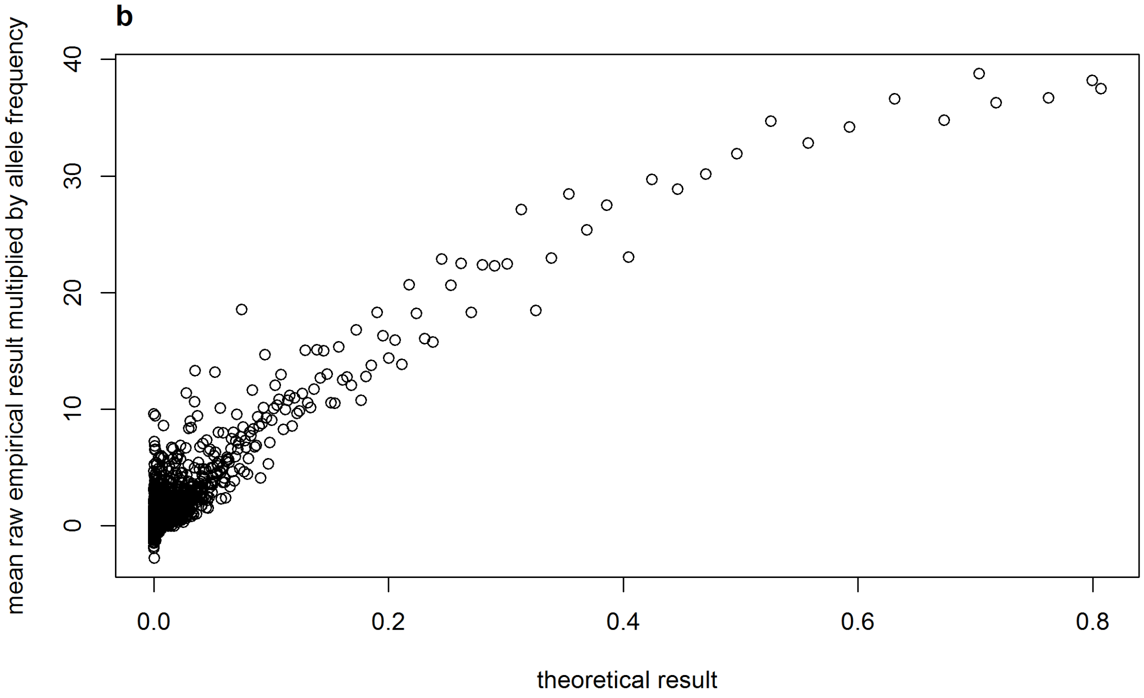

Supplementary Figure S17: Additional simulation with $\bar{S}$ =3, τ=1

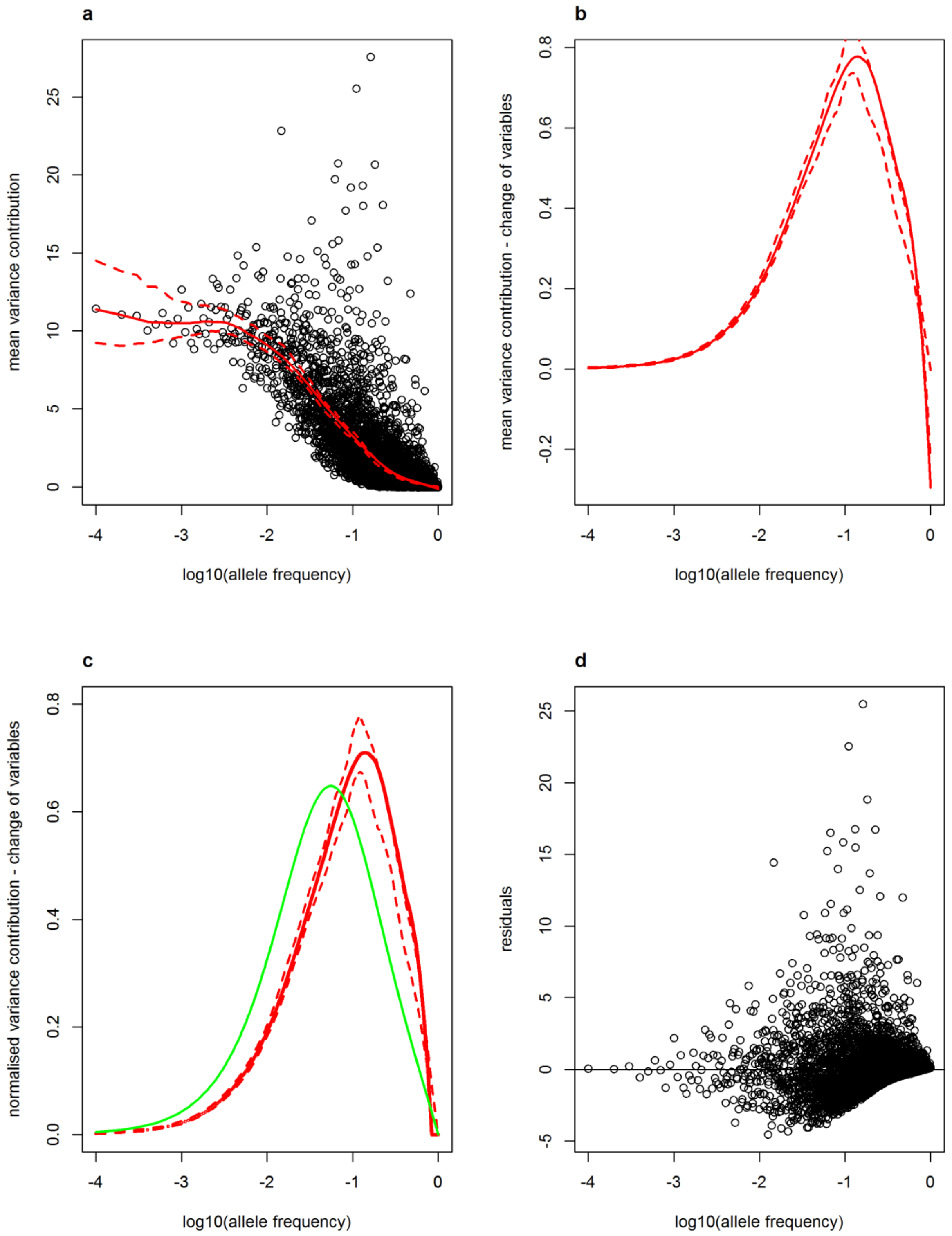

Supplementary Figure S18: Additional simulation with $\bar{S}$ =3, τ=1

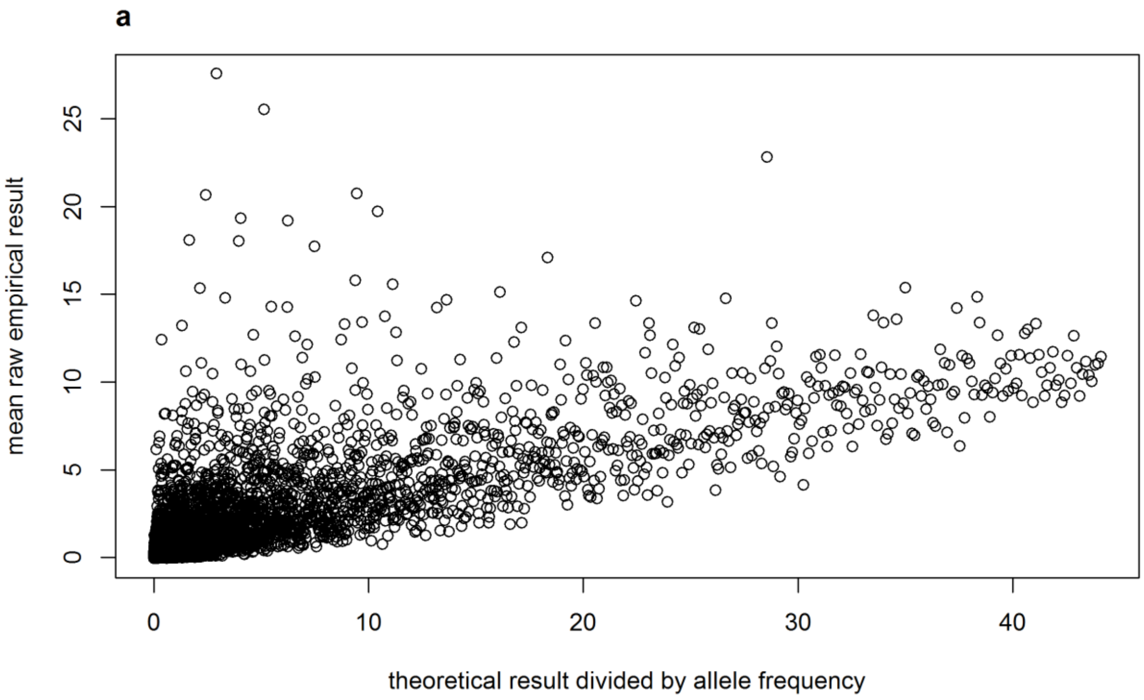

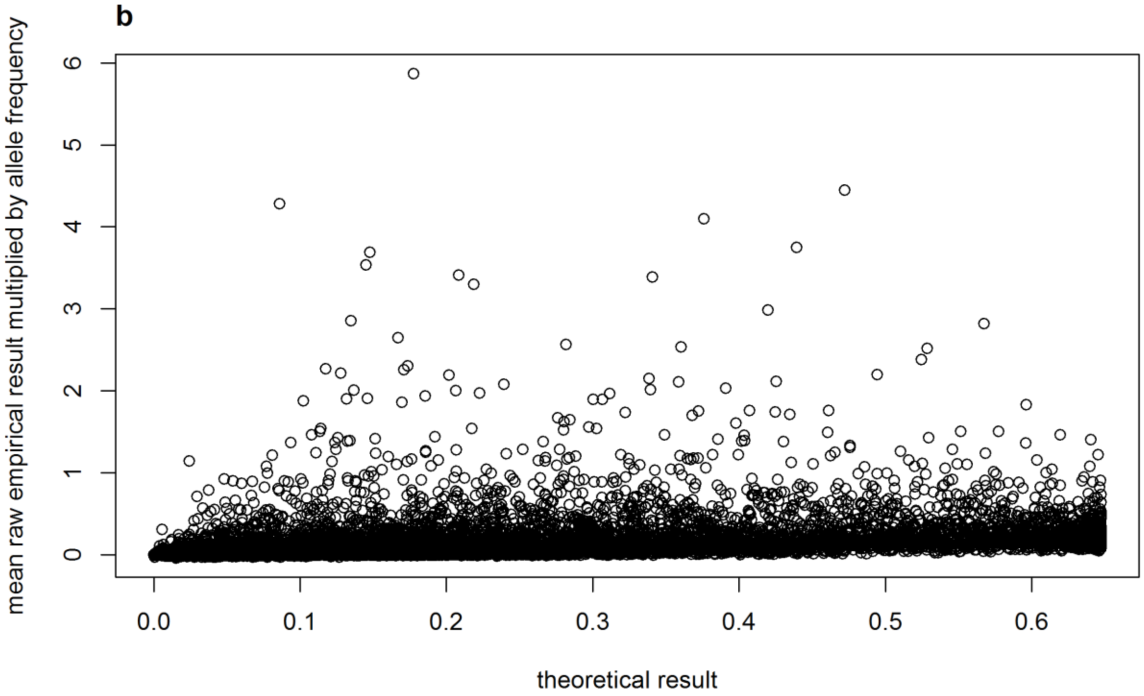

The association between S and z

Supplementary Figure S19: The relationship between S and z by model. Model parameters are that given in Table 1 of the main paper (panels a-h represent models a-h here). The plots were created using random number generation in R[4].

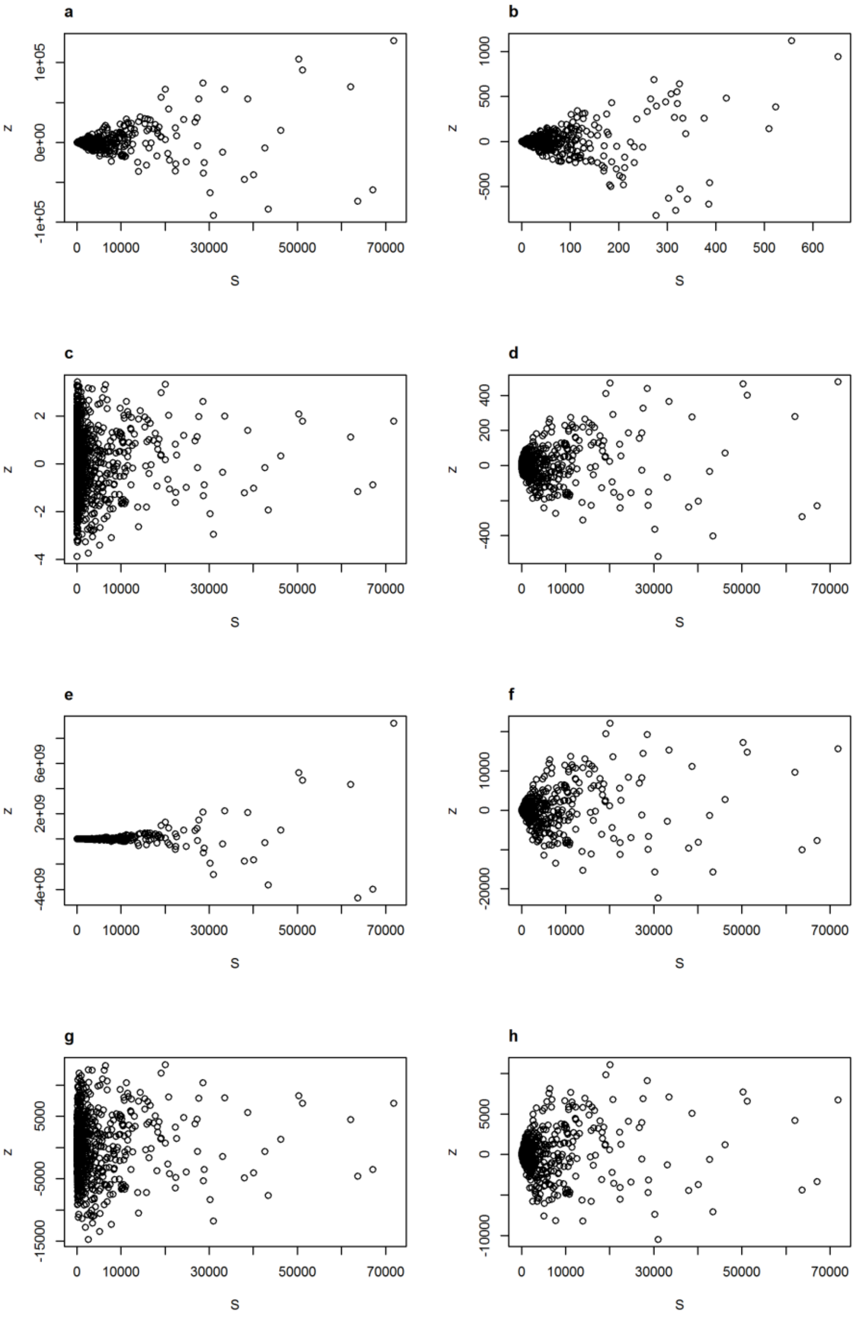

Tables

Model	Mean proportion (2d.p.)	Minimum proportion (2d.p.)	Maximum proportion (2d.p.)
a	0.00	-0.05	0.05
b	0.00	-0.05	0.07
c	0.00	-0.05	0.05
d	-0.00	-0.05	0.04
e	0.00	-0.05	0.06
f	0.00	-0.05	0.05
g	0.00	-0.04	0.04
h	-0.00	-0.07	0.04

Supplementary Table S1: Descriptive characteristics of the proportion of the variance in the trait attributable to covariance terms across runs of each model (parameter assignments as described in Table 1 of main text)

Model	Difference (2.dp) (analytical proportion – empirical proportion)
a	0.00
b	0.20
c	0.00
d	0.01
e	0.00
f	0.01
g	0.13
h	0.02

Supplementary Table S2: Difference in the proportion of the genetic variance due to mutations with frequency less than or equal to 1% between the analytical model which assumes independent loci and the empirical model which includes linkage. Estimates of the difference were obtained by integrating over the values in Figure 4 of the main paper using trapezoidal integration between log10(allele frequency)=-4 to log10(allele frequency)=-2.

References

1. Herrmann E., Maechler M. lokern: Kernel Regression Smoothing with Local or Global Plug-in Bandwidth. R package version 1.1-5. (2013).

2. Eyre-Walker A. Genetic architecture of a complex trait and its implications for fitness and genome-wide association studies. *Proceedings of the National Academy of Sciences* **107**, 1752-1756 (2010).

3. Wolfram Research, Inc. Mathematica. Version 8.0. (Champaign, Illinois, 2010).

4. R Core Team. R: A language and environment for statistical computing. (R Foundation for Statistical Computing, Vienna, Austria, 2014).
